# T cells expressing CD5/CD7 bispecific chimeric antigen receptors with fully human heavy-chain-only domains mitigate tumor antigen escape

**DOI:** 10.1038/s41392-022-00898-z

**Published:** 2022-03-25

**Authors:** Zhenyu Dai, Wei Mu, Ya Zhao, Jiali Cheng, Haolong Lin, Kedong Ouyang, Xiangyin Jia, Jianwei Liu, Qiaoe Wei, Meng Wang, Chaohong Liu, Taochao Tan, Jianfeng Zhou

**Affiliations:** 1 Department of Hematology, Tongji Hospital, Tongji Medical College, Huazhong University of Science and Technology, Wuhan, Hubei 430030 China; 2 Nanjing IASO Biotherapeutics, Nanjing, Jiangsu 210000 China; 3 Department of Pathogen Biology, Tongji Medical College, Huazhong University of Science and Technology, Wuhan, Hubei 430030 China

**Keywords:** Drug development, Immunotherapy

## Abstract

Bispecific chimeric antigen receptor T-cell (CAR-T) therapies have shown promising results in clinical trials for advanced B-cell malignancies. However, it is challenging to broaden the success of bispecific CAR-T therapies to treat refractory/relapse (r/r) T-cell leukemia/lymphoma because targeting multiple T-cell-expressing antigens leads to exacerbated CAR-T cell fratricide and potential safety concerns. Fully human heavy chain variable (FHV_H_) antibodies that specifically target CD5 or CD7 were screened and constructed to CD5/CD7 bispecific CARs. A truncated Epidermal growth factor receptor were integrated into CAR constructs to address safety concerns. To tackle the fratricidal issue of CAR-T cells targeting T-cell-pan marker(s), CRISPR/Cas9-based CD5 and CD7 genes knockout were performed before lentiviral transduction of bispecific CARs. Functional comparison between different bispecific CAR structures: tandem CARs and dual CAR were performed in vitro and in vivo to determine the optimal construct suitable for addressing T-cell malignancy antigen escape in clinical setting. Knockout of CD5 and CD7 prevents fratricide of CD5/CD7 bispecific CAR-T cells, and FHV_H_-derived CD5/CD7 bispecific CAR-T cells demonstrate potent antitumor activity in vitro and in vivo. The fratricide-resistant FHV_H_-derived CD5/CD7 bispecific CAR-T cells have potent antitumor activity against T-cell malignancies, and tandem CARs are more effective than dual CAR in preventing tumor escape in heterogeneous leukemic cells. The meaningful clinical efficacy and safety of tandem CD5/CD7 CAR-T cells deserve to be explored urgently.

## Introduction

CAR-T therapies have achieved great success in clinical trials for advanced B-cell malignancies, with CD19 CAR-T therapy leading to complete remission in many patients suffering from r/r B-cell leukemia/lymphoma.^1,2^ However, a high incidence of relapse with CD19^−^ disease revealed in long-term follow-up data of CD19-targeting immunotherapy underscores the vulnerability of this therapeutic strategy to antigen escape.^3–5^

Similarly, treatment failure or relapse of CAR-T therapies targeting a single antigen to treat refractory/relapse (r/r) T-cell leukemia/lymphoma, a group of heterogeneous malignancies with poorer overall prognosis compared to r/r B-cell malignancies have also been reported in clinical trials.^6–8^ A phase I study (NCT03081910) described by Hill and his colleagues reported that two patients relapsed after infusion of autologous CD5 directed CAR-T cells without planned allo-HSCT due to the underlying CD5^+^ neoplasms.^9^ Pan et al. recently reported encouraging early results from a phase I trial in which patients with r/r acute T-cell leukemia (T-ALL) received allogeneic CD7 directed CAR-T cells. 95% (*n* = 19) of the patients responded to CAR-T therapy, and 90% (*n* = 18) of them achieved complete response (CR). In this trial, one patient experienced treatment failure due to the absence of CD7 expression in bone marrow tumor cells, and one patient developed a CD7 negative relapse.^10^ Tumor recurrence of CD7^-^ was also reported in a recent clinical trial (NCT04004637) using autologous anti-CD7 CAR-T cells to treat CD7^+^ r/r T-lymphocytic leukemia/lymphoma.^11^

Recently, CAR-T therapies targeting multiple B-cell antigens demonstrated the potential to overcome the limitations of CD19 dim or negative expression.^12–14^ However, it is challenging to broaden this strategy to CAR-T therapy for T-cell leukemia/lymphoma. Expression of the targeted T-cell-pan markers on CAR-T cells leads to self-activation, fratricide, and dysfunction of CAR-T cells.^15,16^ The synergistic on-target/off-tumor effect may also contribute to the exacerbated T-cell aplasia, which is less treatable than B-cell aplasia occurring after CAR-T treatment that can be managed with immunoglobulin replacement therapy.

Bispecific CARs based on the classical single-chain variable fragment (scFv) have substantial barriers in clinical application. Firstly, the oversized constructs of dual CAR would affect the packaging of viral vectors and transduction efficiency, thereby impacting the clinical outcomes of bispecific CAR-T therapies.^17,18^ Besides, to ensure the safety of bispecific CAR-T cells to treat T-cell malignancies, switch off systems (e.g., EGFRt, the iCasp9M safety switch) are also need to be integrated into CAR-T cells,^19,20^ which would further increase the size of bispecific CAR constructs. Secondly, intrinsic hydrophobic interaction between V_H_ and V_L_ leads to a strong tendency of scFv to self-aggregate, which may trigger the early exhaustion of CAR-T cells.^21,22^ Thirdly, multiple scFvs in CAR-T cells exacerbates the risk of compromised protein stability, which in turn impairs binding specificity and affinity. Fourthly, murine-derived scFvs further increased the immunogenicity of bispecific CAR-T cells, with greater potential to generate anti-drug antibodies (ADAs) and anti-CAR cytotoxic T lymphocytes (CTLs) leading to poor persistence of CAR-T cells and resulting in limited long-term outcomes.^23,24^ Therefore, replacement of scFv by alternative molecules to construct bispecific CARs for addressing CAR-T therapy failure and/or disease relapse of T-cell malignancies caused by epitope or antigen loss remains a critical area.

To develop a bispecific CAR-T cell strategy to treat T-cell malignancies, we investigated and selected T-cell-pan markers CD5 and CD7 from a well-characterized tumor-specific antigen library, the safety and benefits of which for monovalent CAR-targeting have been established.^9,25,26^ CD5 is regularly expressed on normal T cells and ~85% of T-cell malignancies as well as some B-cell malignancies. It acts as an inhibitory receptor of (TCR) and B-cell receptor (BCR).^27,28^ CD7 is a transmembrane protein expressed on over 90% of lymphoblastic T-cell leukemias and lymphomas, and ~30% of acute myeloid leukemias,^29,30^ but it appears to have limited contribution to the development or function of T cells.^31^ The expression patterns of CD5 and CD7 suggest that they are attractive therapeutic targets for T-cell malignancies.

In this study, we explored the application of V_H_ domains obtained using an in-house developed high-quality fully human V_H_ phage display library to construct CD5/CD7 bispecific CARs and developed fratricide resistance of CD5/CD7 bispecific CAR-T cells. Tandem CAR (Tan CAR) and dual CAR are two major form of bispecific CAR structure currently.^32^ Therefore, functional comparison between tandem CARs and dual CAR were performed and the results revealed that Tan CAR are more suitable for constructing CD5/CD7 bispecific CAR-T cells, which performed better in controlling leukemic cells with heterogeneous antigen expression.

## Results

### Expression of CD5 and CD7 on T-cell surface leads to the fratricide of CD5/CD7 bispecific CAR-T cells

A critical issue of concern is whether normal T cells transduced with CAR lentivirus targeting T-cell-pan marker(s) can resist fratricide to ensure CAR-T cell function. To test whether normal T cells can be redirected to recognize malignant T cells with CD5/CD7 bispecific CAR, activated human T cells were transduced with CAR containing lentivirus directly. In contrast to control CD19 CAR-T cells, the T cells expressing bispecific CAR were associated with poor viability and failed to expand (Supplementary Fig. 1a). Expression of CAR molecules in T cells can be detected by EGFRt antibody instead of recombinant CD5 or CD7 proteins (Supplementary Fig. 1b). Bispecific CAR-T cells showed decreased expression intensity of CD5 and CD7 antigens while significantly higher expression levels of apoptotic related markers (Supplementary Fig. 1c, d). These are consistent with the reported results that CD5 or CD7 targeting CAR-T cells with no antigen blockage are subject to fratricide.^15,16,33^

### Knockout of CD5 and CD7 did not change the biological characteristics of T cells

**Fig. 1:**
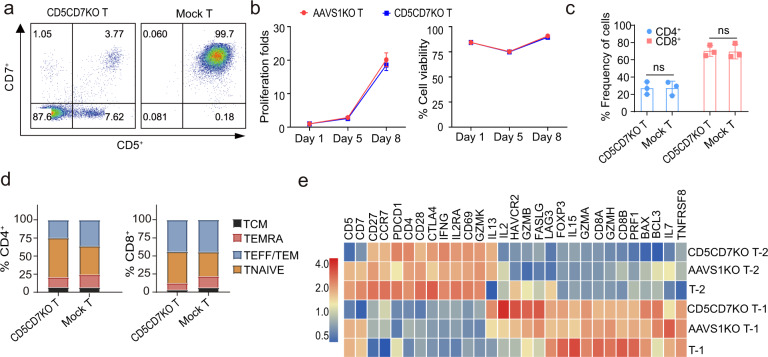
Disruption of CD5 and CD7 genes in T cells with CRISPR/Cas9 system. **a** Representative dot plots showing expression of CD5 and CD7 in CD5CD7KO T and mock T cells determined 3 days post electroporation with Cas9 protein, CD5 sgRNA-7, and CD7 sgRNA-85 using flow cytometry. **b** Proliferation and cell viability of CD5CD7 knockout T cells. Knockout of Adeno Associated Virus Integration Site 1(*AAVS1*), a safe harbor locus was used as a control. **c** The populations of CD4^+^/CD8^+^ T cells in CD5CD7KO T and mock T cells. The data represents mean ± SD for three donors. ns, no significance, two-way ANOVA. **d** The frequencies of effector and effector memory (TEFF/TEM; CCR7^−^ CD45RA^−^), terminally differentiated effector memory (TEMRA; CCR7^−^ CD45RA^+^), naïve (TNAIVE; CCR7^+^ CD45RA^+^), and central memory (TCM; CCR7^+^ CD45RA^-^) in CD5CD7KO T and mock T cells were measured using flow cytometry on days 7–10. The data represents the average from three donors. **e** Heatmap of selected genes related to T-cell activation, exhaustion, cytokine production, differentiation, and function-related genes

Sustained expansion and activity of CD5/CD7 CAR-T cells require disruption of *CD5* and *CD7* genes in T cells, however, whether knockout of CD5 and CD7 affect the biological characteristics of T cells need to be verified. Therefore, CRISPR/Cas9 mediate CD5 and CD7 genes disruption were performed before CAR containing lentivirus transduction in this study. CD5-specific gRNA-7 was derived from our published data,^34^ and CD7-specific gRNA-85 was obtained from the preclinical study reported by Gomes-Silva et al (Supplementary Table 1).^16^ Electroporation of Cas9 protein, CD5 gRNA-7, and CD7 gRNA-85 leads to effective disruption of *CD5* and *CD7* genes, resulting in loss of CD5 and CD7 expression on the surface of T cells (Fig. 1a), without altering the proliferation, viability, CD4/CD8 ratio, differential phenotype (Fig. 1b-d) and functional-related genes in T cells (Fig. 1e). The Venn diagram showed that 13089 and 13236 genes were co-expressed by three groups respectively (Supplementary Fig. 2a). Significantly differentially expressed genes (DEGs) in three groups of two donors were shown by Volcano plots (Supplementary Fig. 2b).

### Manufacturing of CD5/CD7 bispecific CAR-T cells

In a previous study, we obtained an FHV_H_ domain (CD5-FHV_H_61) that showed comparable CD5 targeting ability with murine-derived H65 scFv.^34^ Through three rounds of beads panning, we successfully enriched and obtained four phage V_H_ clones (FHV_H_1–4) that can bind CD7 antigen specifically (Supplementary Fig. 3 and Table 2). FHV_H_1–4 and TH69 scFv as a control were constructed into the CAR BBz lentiviral vector (Supplementary Fig. 4a). Functional assays were performed to compare FHV_H_1–4 with TH69 CAR-T cells. The results suggested that FHV_H_3 CAR-T cells had comparable cytolytic effects on CD7^+^ tumor cells with TH69 CAR-T cells (Supplementary Fig. 4b, c). To test whether CD5 and CD7 targeting V_H_ domains are suitable to construct bispecific CARs, the candidate V_H_ domains of CD7 were connected with CD5-FHV_H_61 through a flexible (GGGGS) × 3 linker and cloned to the CAR BBz lentivirus vector for subsequent functional comparison. The results showed that CD5-FHV_H_61-CD7-FHV_H_3 and CD7-FHV_H_3-CD5-FHV_H_61 had optimal specificity and tumor-killing capability (Supplementary Fig. 5).

**Fig. 2:**
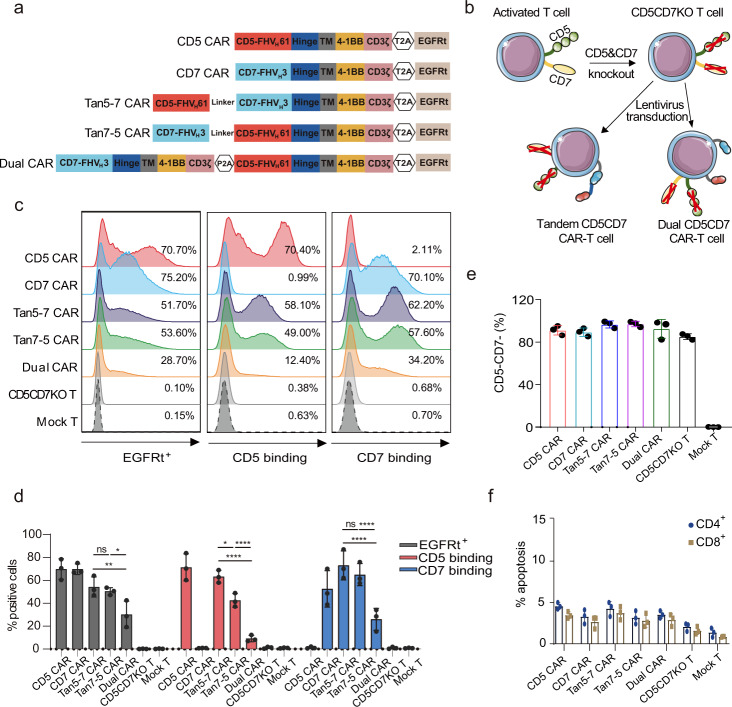
CD5/CD7 bispecific CARs were successfully expressed on the surface of CD5CD7KO T cells. **a** Schematic diagram of the five CARs used in this study: CD5 CAR BBz, CD7 CAR BBz, Tan5-7 CAR BBz, Tan7-5 CAR BBz, and Dual CAR BBz. **b** Cartoon outline of the procedure for generating CD5CD7KO CD5/CD7 bispecific CAR-T cells. This figure was produced using Servier Medical Art (www.servier.com). **c** Representative flow cytometry analysis showing transduction efficiency of CD5CD7KO CAR-T/T and mock T cells on day 7. **d** Quantification and statistical analysis of the data in **c**. The results are displayed as mean ± SD (*n* = 3), *p < 0.05, **p < 0.01, ****p < 0.0001, ns no significance, two-way ANOVA. **e** Flow cytometry analysis of CD5 and CD7 expression on the surface of CD5CD7KO CAR-T/T and mock T cells on day 7. The data represents mean ± SD for three donors. **f** The basal apoptosis of CD5CD7KO CAR-T/T and mock T cells. The results are displayed as mean ± SD (*n* = 3)

To test the tumor-killing efficacy of CD5/CD7 bispecific CARs and single-target CARs, and further reveal the differences between Tan CAR and dual CAR, CD5 CAR BBz, CD7 CAR BBz, Tan5-7 CAR BBz, Tan7-5 CAR BBz, and Dual CAR BBz were constructed (Fig. 2a). Activated T cells were electroporated with ribonucleoprotein (RNP) and transduced with CAR containing lentivirus 24 h later (Fig. 2b). CAR expression was detected by EGFRt antibody, recombinant CD5, and CD7 protein on day 7. Tan5-7 CAR, Tan7-5 CAR, and Dual CAR can be expressed on the surface of CD5CD7KO T cells successfully and can be detected by recombinant CD5 or CD7 protein (Fig. 2c), however, the frequency of EGFRt^+^ cells and the binding rate of CD5 or CD7 protein were significantly lower in Dual CAR-T cells than in Tan5-7 or Tan7-5 CAR-T cells (Fig. 2d). The inferior transduction efficiency may be related to the oversized CAR fragment in Dual CAR-T cells (Tan5-7 CAR BBz: 2589 bp, Tan7-5 CAR BBz: 2589 bp, and Dual CAR BBz: 3384 bp; size for CAR-T2A-EGFRt). CAR-T cells were able to eliminate residual CD5^+^ and/or CD7^+^ cells in all groups (Fig. 2e). Moreover, there was no obvious apoptosis in all CAR-T cell groups with genetic disruption of *CD5* and *CD7* genes (Fig. 2f).

### CD5/CD7 bispecific CAR-T cells demonstrate dual specificity and exhibited potent cytotoxicity against malignant T-cell lines in vitro

**Fig. 3:**
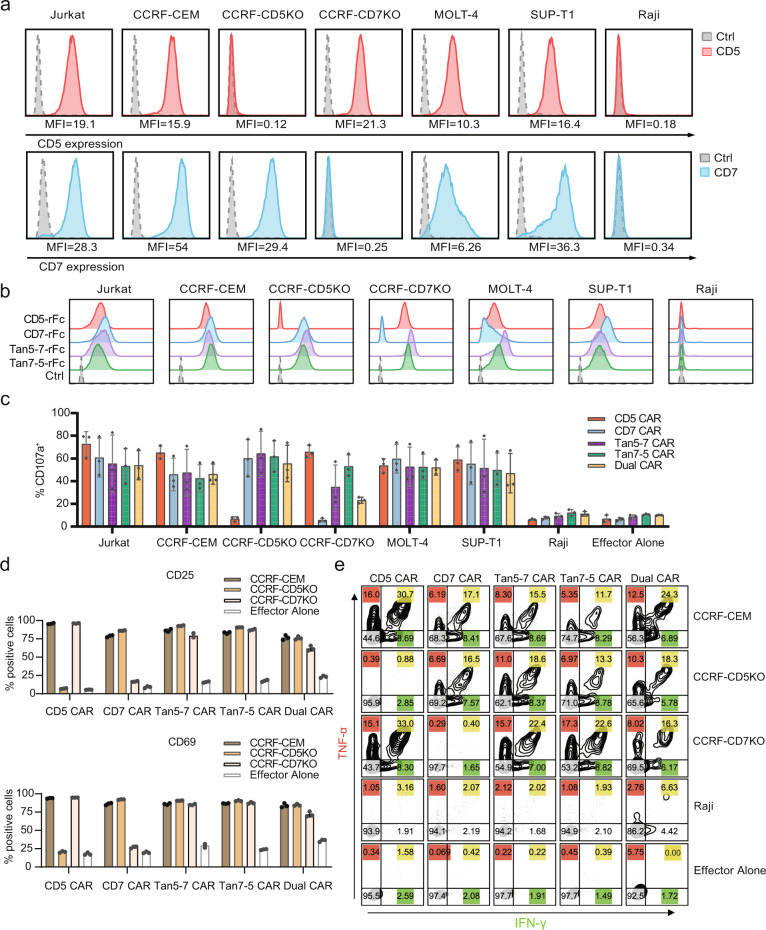
CD5/CD7 bispecific CAR-T cells can be activated by CD5^+^ and/or CD7^+^ cells. **a** Surface expression of CD5 (red solid histograms) and CD7 (blue solid histograms) in T-ALL and T-lymphoma cell lines in comparison with isotype control (dotted line gray histograms) measured using flow cytometry. **b** Cell lines were stained with CD5-rFc, CD7-rFc, Tan5-7-rFc, Tan7-5-rFc antibodies, and isotype control, followed by PE-conjugated anti-rabbit IgG antibody, then analyzed using flow cytometry. **c** The degranulation assay of five CARs. The results are displayed as mean ± SD for three donors. **d** Expression of the T-cell activation markers CD69 and CD25 on CAR-T cells following a 24 h co-incubation with CCRF-CEM, CCRF-CD5KO, or CCRF-CD7KO. Gated on CD8^+^ EGFRt^+^ cells. The data represents mean ± SD (*n* = 3). **e** Representative flow cytometry plots showing intracellular cytokine staining for pro-inflammatory cytokines (TNF-α and IFN-γ) after CAR-T cells cocultured with indicated target cells at an E:T ratio of 1:5 for 24 h

To validate the specificity of CD5/CD7 bispecific CAR-T cells, expression levels of CD5 and CD7 on targeted tumor cell lines were detected by flow cytometry (Fig. 3a). Raji cells are CD5^-^CD7^-^ which is a negative control. Jurkat, CCRF-CEM, MOLT-4, and SUP-T1 are T-cell malignancy cell lines that naturally express CD5 and CD7. whereas CCRF-CEM-CD5 knockout (CCRF-CD5KO, CD5^-^CD7^+^) and CCRF-CEM-CD7 knockout (CCRF-CD7KO, CD5^+^CD7^-^) cell lines are generated from CCRF-CEM cells with CD5 or CD7 being disrupted. Knockout of *CD5* gene slightly increased the proliferation of CCRF-CEM cells, while *CD7* gene knockout hindered the expansion of CCRF-CEM cells (Supplementary Fig. 6).

Tandem use of CD5-FHV_H_61 and CD7-FHV_H_3 can specifically recognize single antigen-positive (CD5^-^CD7^+^ or CD5^+^CD7^-^) cells (Fig. 3b and Supplementary Fig 7), though affinity measurement using the Octet96e system showed that the order of connection affects the affinity of CD5/CD7 bispecific antibodies (Supplementary Fig. 8).

Degranulation was a prerequisite for perforin-granzyme-mediated tumor-killing of CAR-T cells, CD5/CD7 bispecific CAR-T cells upregulated CD107a expression after co-incubated with Jurkat, CCRF-CEM, MOLT-4, SUP-T1, CCRF-CD5KO cells, and CCRF-CD7KO cells. After stimulation with CCRF-CD7KO cells, Tan7-5 CAR-T cells showed significantly higher levels of CD107a than Dual CAR-T cells (*P* < 0.05; Fig. 3c). In addition, compared to unstimulated CAR-T cells, CD5/CD7 bispecific CAR-T cells upregulated the T-cell activation markers CD69 and CD25 after stimulation with CCRF-CEM cells, CCRF-CD5KO cells, or CCRF-CD7KO cells (Fig. 3d), and produced pro-inflammatory cytokines tumor necrosis factor-α (TNF-α) and interferon-gamma (IFN-γ) (Fig. 3e). RNA-seq was performed to further characterize the transcriptomic profiles of CD5, CD7, Tan5-7, Tan7-5, and Dual CAR-T cells following interaction with mitomycin C-treated CCRF-CEM cells for 24 h. As shown in Supplementary Fig. 9a, gene enrichment analysis revealed a major difference in transcriptional profiles related to T-cell activation, cytokine production, and cytokine-mediated signal pathways. At the mRNA level, Dual CAR-T cells showed greater upregulation of T-cell activation related genes such as *IL2RA* and *CD69*, while cytokine genes such as *IL-4*, *IL-5*, and *IFNγ*, and T-cell cytolysis associated genes such *GZMB* and *FasL* were significantly expressed in Tan7-5 CAR-T cells (Supplemental Fig. 9b).

**Fig. 4:**
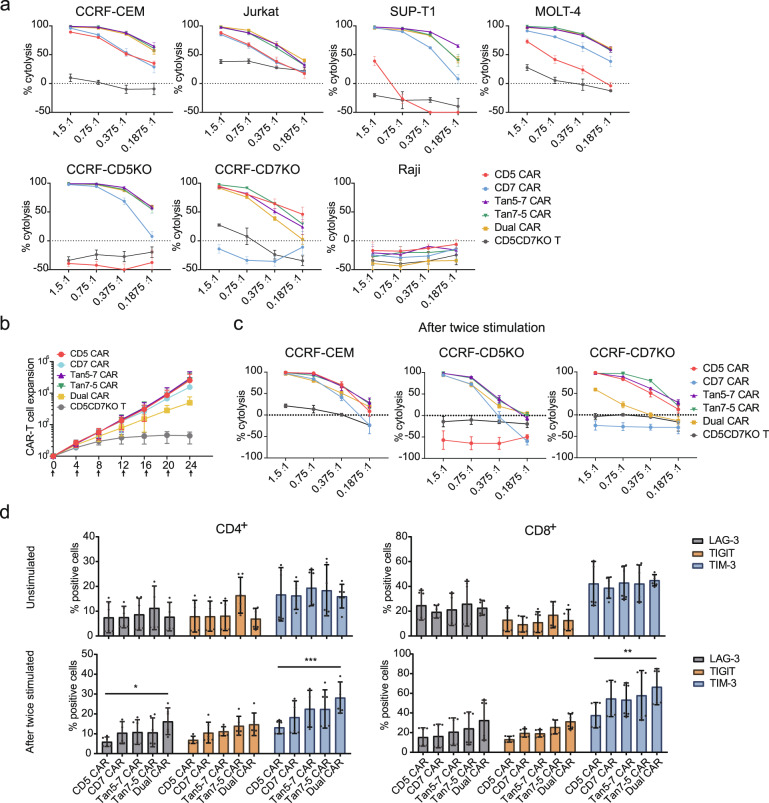
Functional comparison of CD5/CD7 bispecific CAR-T cells was performed in vitro. **a** CD5/CD7 bispecific CAR-T cells specifically kill CD5^+^ and/or CD7^+^ tumor cells. The data indicate mean ± SD (three replicates). **b** Expansion of indicated CAR-T and T cells during multi-rounds of tumor cells stimulation. The data represent mean ± SD from three donors. The X-axis represents different coculture time points of CAR-T/T cells with CCRF-CEM cells and the arrows mean mitomycin C-treated CCRF-CEM cells were added at indicated time. **c** Cytolysis assay of CAR-T/T cells after two rounds of stimulation by mitomycin C-treated CCRF-CEM cells. CAR-T/T cells were remixed with tumor cells at indicated ratios (CAR^+^% to tumor cells) to evaluate the cytotoxicity by luciferase-based assay. The data represent mean ± SD (*n* = 3). **d** CAR-T cells were stained with antibodies against LAG-3, TIGIT, and TIM-3, respectively, before and after two stimulations by mitomycin C-treated CCRF-CEM. The results are displayed as mean ± SD (*n* = 3). *p < 0.05, **p < 0.01, ***p < 0.001, two-way ANOVA

Next, the cytotoxicity of CD5/CD7 bispecific CAR-T cells was assessed using a luciferase-based assay. CD5/CD7 bispecific CAR-T cells selectively killed tumor cells in a dose-dependent manner, but not CD5^-^CD7^-^ Raji (Fig. 4a), demonstrating that CD5/CD7 bispecific CAR-T cells maintain good specificity. CAR-T cell proliferation in the presence of continuous exposure to target antigens is critical for eradicating large tumor burdens in clinical. To this end, we simulated the antigen stimulation situation and assessed the expansion potential, cytotoxicity, and expression of exhaustion markers of CAR-T cells under multiple cycles of antigen stimulation in vitro. Tan5-7 CAR-T cells exhibited significantly higher proliferation than Dual CAR-T cells (*P* < 0.05) and similar proliferation compared to CD5, CD7, and Tan7-5 CAR-T cells (no significant difference, *P* > 0.05) after six rounds of mitomycin C-treated CCRF-CEM cell stimulation (Fig. 4b). After two rounds of mitomycin C-treated CCRF-CEM cell stimulation, Tan5-7, Tan7-5, and Dual CAR-T cells maintained the robust killing ability of both CCRF-CEM and CCRF-CD5KO, but Tan5-7 and Tan7-5 CAR-T cells showed the significantly superior killing ability of CCRF-CD7KO than Dual CAR-T cells (Fig. 4c). Furthermore, the expression of the exhaustion markers LAG-3, TIGIT, and TIM-3 on CAR-T cells before and after two rounds of stimulations was assessed. CD4^+^ Dual CAR-T cells expressed higher levels of LAG-3 and TIM-3 after the second stimulation in CCRF-CEM than CD5 CAR-T cells, and TIM-3 expression levels of CD8^+^ Dual CAR-T cells were also higher than those of CD5 CAR-T cells (Fig. 4d). RNA-Seq was performed to evaluate the overall state of CD5, CD7, Tan5-7, Tan7-5, and Dual CAR-T cells after repeated stimulation by mitomycin C-treated CCRF-CEM cells in vitro. After four rounds of stimulation, transcriptomic profiles related to T-cell exhaustion, cytokine production, and cytokine-mediated signal pathways showed significant enrichment by Go analysis (Supplemental Fig. 9c). Consistent with functional studies, Dual CAR-T showed higher expression of exhaustion-related markers such as PDCD1, CTLA4, TIGIT, and LAG-3 and lower expression of *GZMB* and *FasL* genes which related to T cell killing effects. These RNA-seq results were presented in Supplemental Fig. 9d.

To investigate the functional basis for the major differences in antitumor efficacy between bispecific and single-targeted CAR-T cells, we further examined the formation of the immunological synapse (IS) by these CARs. Immunological synapse is a discrete structural entity, forms after the ligation of specific activating receptors and leads to the cytolysis of tumor cells.^35,36^ In previous studies, F-actin accumulation at the IS and polarization of the microtubule-organizing center (MTOC) were measured to assess whether CAR-T cells have a functional advantage.^37,38^ Compared with single-targeted CAR-T cells, bispecific CAR-T cells showed significantly higher levels of F-action expression (Supplementary Fig. 10a). Similarly, a higher level of MTOC polarization to the IS in bispecific CAR-T cells as reflected by the reduced distance between MTOC and IS (Supplementary Fig. 10b), indicated that more stable IS structures were produced in bispecific CAR-T cells.

### CD5/CD7 bispecific CAR-T cells exhibit robust antitumor activity in vivo

**Fig. 5:**
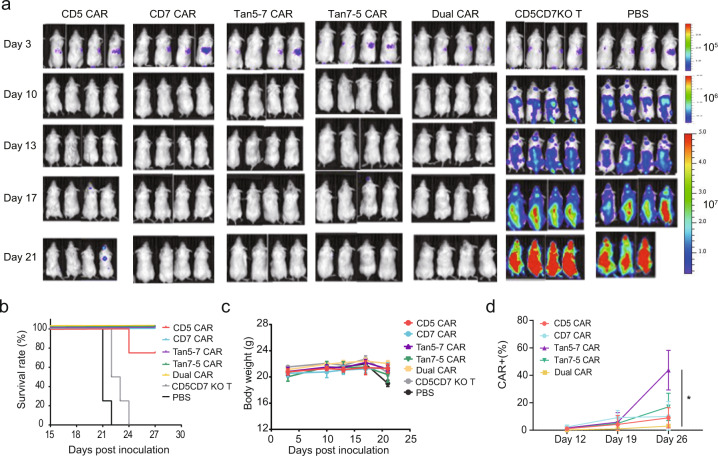
The antitumor activity of CD5/CD7 bispecific CAR-T cells in vivo. **a** Tumor growth and staging were monitored via bioluminescence imaging (BLI). **b** Overall Kaplan–Meier survival curve (log-rank test). Mice treated with CAR-T cells showed significantly increased survival (*P* < 0.0001) compared with those of CD5CD7KO T and PBS-treated groups. **c** Body weight curve. The results are shown as mean ± SEM (*n* = 4). **d** Relative frequency of CAR^+^ T cells (CD45^+^ EGFRt^+^) in peripheral blood of indicated CAR-T-treated mice groups on days 12, 19, and 26, respectively (*n* = 4). *p < 0.05, two-way ANOVA

After observing the superior function of CAR-T cells, we evaluated the in vivo efficacy using cell line derived xenograft (CDX) models established by grafting CCRF-CEM-ffLuc cells through tail vein injection. We tested the ability of CAR-T cells administered on day 4 post-engraftment to suppress leukemia progression. All CAR-T treated groups observed the delayed tumor progression and significantly prolonged survival time of tumor-bearing mice (Fig. 5a, b), with no significant difference in mouse body weight between groups (Fig. 5c). The relative percentage of CD45^+^ EGFRt^+^ T cells in the peripheral blood of CD5/CD7 bispecific CAR-T-treated groups on days 12, 19, and 26 were detected using flow cytometry. The frequency of Tan5-7 CAR-T cells was significantly higher than that of Dual CAR-T cells on day 26 (*P* < 0.05; Fig. 5d).

Encouraged by the efficacy of CAR-T cells in the T-ALL mouse model, we evaluated whether administration of EGFRt-specific antibody cetuximab could eliminate the EGFRt-expressing T cells in vitro and in vivo. As shown in Supplementary Fig. 11a, EGFRt-expressing T cells could be lysed by cetuximab-mediated NK cells. Further, NCG mice have intravenously injected with 1 × 10^6^ SUP-T1 cells on day (−4) and 3.35 × 10^5^ CAR-T cells on day 0, then PBS, cetuximab, and/or NK cells were administrated intraperitoneally on days 1, 4, 7, and 10 as shown in Supplementary Fig. 11b. Mice treated with CAR-T cells (G2) and CAR-T combined with NK cells groups (G3: CAR-T + NK) showed significantly lower tumor burden and there is no significant difference in mouse body weight between groups (Supplementary Fig. 11c, d). Collectively, these data suggest that cetuximab can effectively deplete EGFRt-expressing CAR-T cells.

### Tandem CAR-T cells outpace Dual CAR-T cells in a heterogeneous xenograft tumor model

**Fig. 6:**
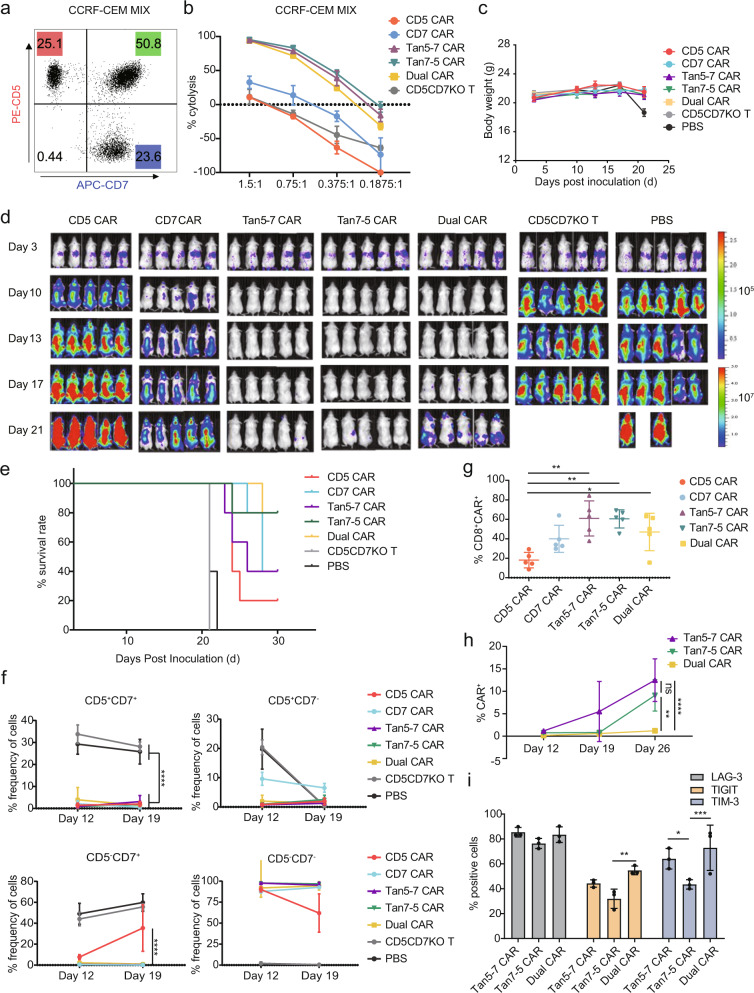
CD5/CD7 bispecific CAR-T cells mitigate single antigen escape. **a** Flow cytometry dot plot showing the CCRF-CEM MIX cell components used to establish heterogeneous tumor model in mice. **b** The killing ability of CAR-T/T cells for CCRF-CEM MIX cells was determined by luciferase-based cytotoxicity assay after 24 h incubation. The data indicates mean ± SD, three independent experiments were performed. **c** Body weight curve. The results are shown as mean ± SEM (*n* = 5). **d** Tumor growth and staging were monitored via bioluminescence imaging (BLI). **e** Overall Kaplan–Meier survival curve (log-rank test). Mice treated with CAR-T cells showed significantly increased survival (*P* < 0.001) compared with those of CD5CD7KO T and PBS-treated groups. **f** Flow cytometry analysis of CD5 and CD7 expression on the surface of CD45^+^ cells in peripheral blood of mice on days 12 and 19 (*n* = 5). ****p < 0.0001, two-way ANOVA. **g** Proportion of CD8^+^ CAR^+^ T cells (CD8^+^ EGFR^+)^ in peripheral blood of NCG mice on day 19 (*n* = 5). *p < 0.05, **p < 0.01, two-way ANOVA. **h** Proportion of CAR^+^ T cells (CD45^+^ EGFR^+^) in peripheral blood of CD5/CD7 bispecific CAR-T cell-treated NCG mice on days 12, 19, and 26, respectively (*n* = 3). **p < 0.01, ****p < 0.0001, ns, no significance, two-way ANOVA. **i** The *p*ercentage of LAG-3^+^, TIGIT^+^, and TIM-3^+^ cells in the peripheral blood of NCG mice collected on day 26 was quantified and statistically analyzed. The results are shown as mean ± SD (*n* = 3). *p < 0.05, **p < 0.01, ***p < 0.001, two-way ANOVA

To assess the ability of CD5/CD7 bispecific CAR-T cells to prevent antigen escape from heterogeneous tumors in vivo, NCG mice were transplanted with 1 × 10^6^ CCRF-CEM MIX-ffLuc (CCRF-CEM-ffLuc:CCRF-CD5KO-ffLuc:CCRF-CD7KO-ffLuc ≈ 2:1:1) (Fig. 6a). Before CAR-T cell infusion, their cytotoxicity to CCRF-CEM-MIX was measured. There was no significant difference among Tan5-7, Tan7-5, and Dual CAR-T cells of their in vitro killing capacity to CCRF-CEM MIX assessed using a luciferase-based assay (Fig. 6b). Each mouse in the CAR-T treated groups was infused with 4 × 10^6^ CAR^+^T cells via tail injection on day 4, and their body weight, tumor burden, and survival time were monitored. There was no significant difference in weight change between the groups (Fig. 6c). CD5/CD7 bispecific CAR-T cells are protective in the heterogeneous xenograft tumor model. Tan5-7 and Tan7-5 CAR-T cells eliminated neoplastic cells in mice and significantly prolonged the suppression of leukemia compared with Dual CAR-T cells (Fig. 6d). All mice treated with CD5 CAR-T and CD5CD7KO T cells progressed steadily, while CD7 CAR-T cells were able to temporarily inhibit neoplastic cells but failed to control the progression of leukemia due to the loss of CD7 antigen on tumor cells (Fig. 6d). All CARs were effective in prolonging the survival of tumor-bearing mice (*P* < 0.001; Fig. 6e).

CD5 and CD7 expression on the surface of CD45^+^ cells in the peripheral blood of mice were measured using flow cytometry to assess the effect of CAR-T treatment on heterogeneous leukemia cells. Compared with single CAR targeting T cells, CD5/CD7 bispecific CAR-T cells conferred robust protection against the accumulation of single antigen-positive leukemia cells in the peripheral blood of mice (Fig. 6f and Supplementary Fig. 12), and the proportion of CD8^+^ CAR-T cells in the peripheral blood of mice in the CD5/CD7 bispecific CAR-T treated groups were significantly higher than those in the CD5 CAR-T treated group on day 19 (Fig. 6g).

To evaluate the expansion and persistence of CD5/CD7 bispecific CAR-T cells, the percentage of CD45^+^ EGFRt^+^ cells in the peripheral blood of mice was determined using flow cytometry on days 12, 19, and 26. The results showed that Tan5-7 and Tan7-5 CAR-T cells had more durable expansion in vivo compared to Dual CAR-T cells (Fig. 6h). In addition, quantification and statistical analysis results of the proportion of LAG-3^+^, TIGIT^+^, and TIM-3^+^ cells in CD5/CD7 bispecific CAR-T cells in the peripheral blood of NCG mice collected on day 26 suggested that Tan7-5 CAR-T cells exhibited lower levels of LAG-3 and TIM-3 than Dual CAR-T cells and lower levels of TIM-3 than Tan5-7 CAR-T cells (Fig. 6i).

## Discussion

Although T-cell-pan marker(s) are attractive therapeutic targets for T-cell malignancies, it is critical to solving the fratricidal problem of CAR-T cells targeting T-cell-pan marker(s) to enable CAR-T cells with the capacity to expand and not compromise their tumor-killing function. Preliminary data from a clinical trial of CD5 CAR-T therapy using murine-derived scFv showed a favorable safety profile, but the efficacy was modest (44% of patients achieved objective responses) and not durable,^9^ probably due to CAR-T cell fratricide. Alotaibi et al. demonstrated that functional blockade of CD5 induced an increased level of T-cell activation and its enhanced antitumor activity.^39^ Chun et al. also reported that CD5KO boosted the antitumor activity of CAR-T cells by enhancing the activation and proliferation mediated by CAR molecules.^40^ Similarly, CAR-mediated T-cell fratricide has been observed in CD7 CAR-T cells. Zhang et al. established a strategy by anchoring CD7 in the ER and/or Golgi to overcome CAR-T cell fratricide.^41^ Several clinical trials have shown promising results with “off-the-shelf” CAR-T cells targeting CD7 obtained by knocking out *TRAC* and *CD7* through CRISPR/Cas9 gene editing technology to avoid graft-versus-host disease and fratricide.^25,26^ In clinical trials of CAR-T therapies targeting CD5 or CD7, complete T-cell dysplasia has not been observed. Georgiadis et al. considered the issue of antigen escape on the T-cell malignancy populations and established a strategy of CD3 and CD7 CARs combinational therapy to treat T-cell malignancies by genomic editing to remove TCR/CD3 and CD7.^42^ However, only a small subset of T-ALL expresses CD3, limiting the application of CD3 CAR-T therapy for T-ALL. In addition, there is a risk that TCR/CD3-specific CAR-T cells promote cross-linking of TCRs leading to T-cell activation and strong rejection responses.^43^ The strategy of mixing two different monovalent CAR-T cells is challenging for product manufacture and requires more specialist manpower, time, and financial resources than the preparation of bispecific CAR-T cells. The possible disproportionate expansion of different CAR-T cells in vivo may have an impact on efficacy.

In this study, we developed fratricide-resistant CD5/CD7 bispecific CAR-T therapies derived from fully human CD5 and CD7 V_H_s by using CRISPR/Cas9 to disrupt the CD5 and CD7 genes in T cells before transduction. Knockout of CD5 and CD7 genes did not change T-cell phenotype and CD4/CD8 ratio. CD5CD7KO T cells expressing either monovalent FHV_H_-derived CAR or CD5/CD7 bispecific FHV_H_-derived CAR could effectively kill target cells and showed antigen-specific proliferation. Results from preclinical studies show that anti-CD33 V_H_ and anti-B-cell maturation antigen (BCMA) V_H_ (FHV_H_33) function similarly to murine scFv-derived benchmarks in vitro and in vivo, highlighting the broad promise of FH single-domain antibody-derived CAR-T therapies. Several preclinical studies of FH single-domain antibody-derived CAR-T therapies showed similar in vitro and in vivo functions to those of murine scFv-derived benchmarks, such as CD33-targeting V_H_ (CAR33V_H_) and B-cell maturation antigen (BCMA)-targeting V_H_ (FHV_H_33),^44,45^ and bispecific FHV_H_-derived CAR-T therapy targeting both prostate-specific membrane antigen (PSMA) and mesothelin (MSLN) also showed favorable perspectives for the treatment of solid tumors.^46^ Compared with scFv binding domains, FH V_H_s have distinct advantages in constructing multispecific CARs. First, the absence of V_L_ and the short peptide linked to V_H_ simplifies the structural design of CARs. Second, unlike the potential pairings between V_L_ and V_H_ domains of different scFvs,^47^ the strict monomeric behavior of heavy-chain-only domains provides important benefits for maintaining the protein stability and the affinity for the specific antigen of multispecific CARs. Third, the small size of heavy-chain-only domain (about half the size of scFv) offers a potential steric advantage in accessing cryptic antigenic epitopes compared to the larger scFvs.^18,48^ Finally, the smaller size and potentially lower immunogenicity of FHV_H_s have a role in addressing the problems caused by human anti-mouse (HAM) immune responses. Taken together, the small size, strict monomeric behavior, and potentially lower immunogenicity of FH heavy-chain-only domains make them ideal building blocks for multispecific CAR constructs.

The tandem CAR (Tan5-7 CAR and Tan7-5 CAR) and dual CAR constructs integrating the original FH anti-CD5 V_H_ domain (CD5-FHV_H_61) and anti-CD7 V_H_ domain (CD7-FHV_H_3) used in this study were able to safeguard against the impacts of antigen escape regardless of either CD5 or CD7 expression loss. Comparative in vitro and in vivo functional results of Tan5-7 CAR, Tan7-5 CAR, and Dual CAR suggest that tandem CARs are more beneficial in preventing relapse due to antigen escape.

Partly due to the smaller DNA fragment that relieves the viral packaging and transduction payloads (reduced by ~23% in DNA length for tandem CARs compared to Dual CAR), the transduction efficiency of tandem CARs on CD5CD7KO T cells was significantly higher than that of Dual CAR. Despite undergoing two rounds of antigen stimulation, Tan5-7, and Tan7-5 CAR-T cells maintained cytolytic stamina and showed significantly better lysis of CD7^−^ tumor cells than Dual CAR-T cells. Upon repeated interactions with heterogeneous tumor cells in vivo, Tan5-7 and Tan7-5 CAR-T cells exhibit robust expansion and lower levels of exhaustion markers than Dual CAR-T cells, which in turn significantly inhibit heterogeneous neoplastic progression in tumor-bearing mice and perhaps point to potential superiority for control of antigen escape in the clinic. The order of anti-CD5 V_H_ and anti-CD7 V_H_ in the tandem CAR constructs does not appear to have a substantial impact on CAR-T cell function. While Tan7-5 CAR-T cells showed a lower TIM-3 expression level than Tan5-7 CAR-T cells (Fig. 6i), the underlying mechanisms need to be explored. Furthermore, Tan5-7 and Tan7-5 antibodies do not bind CD5^-^CD7^-^ cells of multi-tissue origin, proving to maintain favorable specificity and be suitable for further development.

Although complete T-cell aplasia was not observed in clinical trials of CD5 or CD7 CAR-T therapy, the risk of severe T-cell immunodeficiency resulting from simultaneous targeting of two T-cell-pan markers remains a major concern. Therefore, EGFRt was incorporated in the CD5/CD7 bispecific CAR constructs as a safety switch to abrogate or reduce the risk of eradicating host T lymphocytes and other serious toxicities. A further strategy is to bridge allo-HSCT after achieving deep molecular remission with CD5/CD7 bispecific CAR-T therapy to reconstitute patients’ immune systems and subsequently achieve long-term leukemia-free survival.

In conclusion, we have demonstrated that CD5/CD7 bispecific CARs based on FH V_H_s are easily designed, expressed, and functional. In contrast to Dual CAR, Tan5-7 and Tan7-5 CARs present effective and clinically applicable solutions to the challenge of antigen heterogeneous expression in neoplastic populations.

## Materials and methods

### Plasmid construction

CD5-specific FHV_H_ domain and CD7-specific FHV_H_ domains were constructed into second-generation CAR structure containing a CD8α hinge/transmembrane region, 4-1BB co-stimulatory domain, and intracellular CD3ζ. EGFRt was linked with the CAR fragment through a T2A sequence to facilitate the detection of CAR-expressing cells and allow for further translational and clinical research. In the tandem V_H_ CAR and IgG protein vector constructs, the two V_H_s were linked by a flexible (GGGGS) × 3 linker with either the CD5-FHV_H_61 at the N-terminus (CD5-FHV_H_61-CD7-FHV_H_3, termed Tan5-7) or the CD7-FHV_H_3 at the N-terminus (CD7-FHV_H_3-CD5-FHV_H_61, termed Tan7-5). The dual CAR construct contains two individual CARs (CD7 CAR BBz and CD5 CAR BBz) linked by the P2A sequence, enabling the expression of CD7 CAR and CD5 CAR on the surface of a single T cell, simultaneously.

### Cell lines

Jurkat, CCRF-CEM, MOLT-4, and SUP-T1 cells derived from acute T-cell leukemia or T-cell lymphoma are CD5^+^CD7^+^ cell lines. Raji (Burkitt’s lymphoma), RPMI-8226 (multiple myeloma), and NALM-6 (acute B-lymphocytic leukemia) cell lines were CD5^-^CD7^-^. These cells were cultured in RPMI-1640 medium containing 10% fetal bovine serum (FBS; Thermo Fisher, Waltham, MA, USA). HCT-116 (colorectal carcinoma), HEPG2 (hepatocellular carcinoma), MDA-MB-468 (breast adenocarcinoma), OVCAR3 (ovarian adenocarcinoma), NCI-H460 (large cell lung cancer), 293CT (embryonic kidney), KATO III (gastric carcinoma), and PANC-1 (pancreatic epithelioid carcinoma) were cultured in DMEM (Corning, NY, USA) medium containing 10% FBS. All cell lines were verified before use. CCRF-CD5KO and CCRF-CD7KO were generated by knockout of *CD5* and *CD7* genes respectively in CCRF-CEM cells.

### Phage display to generate CD5 and CD7-specific FHV_H_ domains

CD5 and CD7-specific V_H_ domains were screened from the FH heavy-chain-only phage display antibody library (IMARS; Nanjing IASO Biotherapeutics, Nanjing, China) through optimal protein panning. The specificity of outputs was further assessed by ELISA and flow cytometry.

### Manufacturing of CD5CD7KO bispecific CAR-T cells

Human donor peripheral blood leukocytes from healthy donors were used for in vitro and in vivo CAR-T/T functional validation. The study protocol was approved by the Institutional Review Board of Tongji Hospital, Tongji Medical College, Huazhong University of Science and Technology. Appropriate informed consent was obtained from all donors before specimen collection, following the Declaration of Helsinki. Peripheral blood mononuclear cells (PBMCs) were isolated from the collected blood leukocytes via density gradient centrifugation using Ficoll-Paque Plus (GE Healthcare, Boston, MA, USA). CD3^+^ T cells were isolated with CD3 microbeads (Miltenyi Biotec, Bergisch Gladbach, Germany) according to the manufacturer’s instructions and were cultured in X-VIVO 15 medium (Lonza, Basel, Switzerland) supplemented with 10% FBS (Thermo Fisher), 100 U/mL IL-2 (Sigma-Aldrich, St. Louis, MO, USA). Dynabeads™ Human T-Activator CD3/CD28 (Thermo Fisher) were used to activate T cells. Genomic disruption of the CD5 and CD7 genes in activated T cell were performed using the Celetrix electroporation system (Celetrix, Manassas, VA, USA). Detailed procedures of electroporation as follows: (1) Pre-warm T cell culture medium at 37 °C for 20 min. (2) CD3/CD28 dynabeads were removed after 18–20 h stimulation, T cells were washed with PBS once and prepared for electroporation. (3) Ribonucleoprotein was prepared immediately before electroporation. 10 μg Cas9 protein (Kactus Biosystems) with 5 μg CD5 sgRNA or 5 μg CD7 sgRNA were incubated respectively at room temperature for 15 min. (4) 2.5 × 10^6^ T cells were resuspended with 20 μl buffer (10 μl buffer A + 10 μl buffer B), then mixed with CD5 and CD7 RNPs. (5) The mixer containing T cells, buffer, CD5 RNP and CD7 RNP was transferred to an electric tube and electroporated at 810 V, 20 ms. (6) Following electroporation, T cells were incubated in X-VIVO 15 supplemented with 10% FBS, 100 U/mL IL-2, 40 ng/mL IL-7 (Novoprotein Scientific) and 50 ng/mL IL-15 (Novoprotein Scientific). (7) Lentivirus transduction: CD5CD7KO T cells were transduced with CAR containing lentivirus at a multiplicity of infection of 2–5 1 day post electroporation.

### Flow cytometry

PE-conjugated mouse anti-human CD5 antibody (clone: UCHT2; BD Pharmingen, San Diego, CA, USA), isotype antibody (clone: MOPC-21/CD7-6B7; BioLegend), and PE-conjugated mouse anti-human CD7 antibody were used to determine the expression level of CD5 and CD7 on different cell lines.

CD5-hFc-Bio (Kactus Biosystems, Shanghai, China), CD7-hFc-Bio (Kactus Biosystems), and PE, FITC, or APC-conjugated EGFR antibody (clone: AY13) were used to detect CAR expression. PE/Cyanine7-conjugated CD4 (clone: A161A1), BV421-conjugated CD8 (clone: 53-6.7), FITC-conjugated CD8 (clone: SK1), APC-conjugated CD45RA (clone: HI100), BV421-conjugated CCR7 (clone: G043H7), APC-conjugated LAG-3 (clone: 7H2C65), BV421-conjugated TIM-3 (clone: F38-2E2), PE/Cyanine7-conjugated TIGIT (clone: A15153G), PE-conjugated anti-rabbit IgG antibody (clone: Poly4064), BV421-conjugated CD69 antibody (clone: FN50), FITC-conjugated CD25 antibody (clone: BC96), PE/Cyanine7-conjugated CD107a (clone: H4A3), and FITC-conjugated CD45 (clone: HI30) antibodies were all purchased from BioLegend to evaluate CAR-T/ T-cell state.

Apoptosis Detection Kit (BioLegend) was used to evaluated the apoptosis of CAR-T/T cells following the manufacturer’s instructions and then subjected to flow cytometry analysis.

The degranulation of CD5CD7KO CAR-T cells was stained by PE/Cyanine7-conjugated CD107a antibody (clone H4A3; BioLegend) after co-incubated with different target cells for 4 h and detected using flow cytometry.

PE-conjugated TNF-α (clone: MAb11, BioLegend) and PE/Cyanine7-conjugated IFN-γ (clone: 4 S.B3, BioLegend) antibodies were used to detect TNF-α and IFN-γ releasement of CAR-T cell as follow: CAR-T cells were incubated with indicated target cells at the ratio of 1:5. Brefeldin A (BioLegend) and monensin (BioLegend) were added 1 h after plating. Cells were permeabilized for 20 min using BD FACS Permeabilizing Solution 2 and stained with TNF-α and IFN-γ antibodies 4 h post-incubation.

These data were acquired with MACS Quant Analyzer 10 (Miltenyi Biotec) and analyzed with FlowJo software version 10 (Tree Star, Ashland, OR, USA).

### Luciferase-based cytolysis assay

Luciferase-based cytotoxicity was performed as previously described. Briefly, CD5CD7KO CAR^+^ T/T cell and target cells (Jurkat, CCRF-CEM, CCRF-CD5KO, CCRF-CD7KO, MOLT-4, SUP-T1, and Raji cells) were seeded in the white opaque plate in triplicate at indicated ratios and incubated at 37 °C in 5% CO_2_ for 24 h. Luminescence detection was using the Steady-Glo® Luciferase assay system (Promega, Madison, WI, USA) according to the manufacturer’s instructions.

### Repeat antigen stimulation assay

For the repeat antigen stimulation assay, on day 0, CCRF-CEM were plated in 6-well plates treated with mitomycin C at a final concentration of 1 µg/mL. On day 1, mitomycin C-treated CCRF-CEM (3 × 10^5^) were washed six times with PBS and then mixed with 3 × 10^5^ viable CD5CD7KO CAR-T/T cells in 24-well plates with X-VIVO 15 medium supplemented with IL-2, different groups of CD5CD7KO CAR-T cells were normalized for CAR expression by adding CD5CD7KO T cells. On day 4, new CCRF-CEM was treated as on day 0, viable CAR-T cells were counted, and 3 × 10^5^ CD5CD7KO CAR-T/T cells from the 24-well plates that expanded were remixed with 3 × 10^5^ mitomycin C-treated CCRF-CEM as on day 1. This process was repeated five times. Fold expansion after each stimulation was calculated as (viable CAR-T/T cells on day 4)/(3 × 10^5^), whereas the cumulative fold expansion was regulated by ([fold expansion_*n*_]) × ([fold expansion_*n*+1_] …).

### Cetuximab-mediated depletion of EGFRt-expressing CAR-T cells

In vitro assay: NK cells derived from healthy donor were used as effector cells. At different cetuximab concentrations, NK cells and EGFRt-expressing CAR-T cells were cocultured at 37 °C, the ratio of NK to CAR-T cells was 5:1, 5:2, and 1:1, CAR-T cells was detected by flow cytometry 4 h later.

In vivo assay: female 5–6 weeks old NCG mice were intravenously injected on day -4 with 1.0 × 10^6^ SUP-T1 cells. CAR-T cells were infused at 3.35 × 10^5^ per mouse on day 0. NK cells were infused at 1.0 × 10^7^ per mouse at day 1 and day 4. Cetuximab was infused at 0.5 mg per mouse at day 1, 4, 7, and 10. Control mice received PBS of the same volume. The tumor burden was evaluated using bioluminescence imaging (BLI) at day 0, 5, 10, and 13. Body mass and survival were monitored every 2 or 3 days.

### Mouse xenograft models

Animal experiments were accomplished by GemPharmatech (Nanjing, China). The protocol and procedures involving the care and use of animals in this study were reviewed and approved by the Institutional Animal Care and Use Committee (IACUC) of GemPharmatech before the experiments began. The animals were handled in accordance with the regulations of the Association for Assessment and Accreditation of Laboratory Animal Care (AAALAC). Researchers from GemPharmatech were blinded in the animal studies. In the cancer model established by CCRF-CEM, 6-week-old female NOD-Prkdc^em26Cd52^Il2rg^em26Cd22^/Nju (NCG) mice were engrafted with 1.0 × 10^6^ CCRF-CEM-firefly luciferase cells via tail injection on day 0. Then, the mice were treated with 4.0 × 10^6^ CAR^+^T or CD5CD7KO T cells via tail injection on day 4, PBS-treated groups were considered as control (*n* = 4 for each group). In heterogeneous cancer model established by CCRF-CEM MIX (CCRF-CEM:CCRF-CD5KO:CCRF-CD7KO ≈ 2:1:1), 6-week-old female NCG mice were engrafted with 1.0 × 10^6^ CCRF-CEM MIX-firefly luciferase cells via tail injection on day 0. Then, the mice were treated with 4.0 × 10^6^ CAR^+^T or CD5CD7KO T cells via tail injection on day 4, PBS-treated groups were considered as control (*n* = 5 for each group). The leukemic burden was evaluated using bioluminescence imaging, body mass and survival were monitored at indicated time points.

### Antibody affinity measurement

Octet96e system (ForteBio, Menlo Park, CA, USA) was used to evaluate the binding affinity of antibodies to CD5 or CD7. Briefly, antibodies were diluted to 20 μg/mL with loading buffer and loaded at ~0.8 nM onto the Biosensors. After a 60 s equilibration phase, the binding kinetics of the CD5 or CD7 antigen were monitored at multiple antigen concentrations (12.5–400 nM). Each concentration was tested for 160 s of association and 300 s of disassociation. The binding kinetics were analyzed using a 1:1 binding site model (Biacore X100 version 2.0; Cytiva, Marlborough, MA, USA).

### Confocal imaging on the planar lipid bilayer

Planar bilayers were formed by using small liposome droplets with clean glass coverslips as described previously. Briefly, biotin-labeled CD5 and CD7 proteins (ACRO system) were added to the bilayer and incubated for 30 min at 37 °C. After they were washed, 1.0 × 10^5^ CAR^+^ T cells of each group were suspended in T-cell medium and incubated with the lipid bilayers for 15 min at 37 °C. Then, the cells were fixed for 30 min with 4% paraformaldehyde at 4 °C. To visualize the immune synapse, F-actin was stained with Alexa Fluor 594-conjugated phalloidin (Abcam) following the manufacturer’s instructions. Subsequently, a FITC-conjugated AffiniPure F(ab)'2 fragment-specific goat anti-mouse IgG antibody (Jackson ImmunoResearch) or FITC-conjugated anti-gamma tubulin antibody (Abcam) was used to stain CARs or MTOCs, respectively. Nuclei were stained with Hoechst (Sigma) for 5 min. Confocal images were acquired by a Leica SP8 confocal laser-scanning microscope. All images were confirmed not to be overexposed by the software. All images were processed and analyzed with LAS X (Leica) and ImageJ (National Institutes of Health).

### Graphs and statistical analysis

Graphs and data analyses were performed using GraphPad Prism Software version 8.3.0. Some of these graphs were obtained and modified from Servier Medical Art. Unless otherwise stated, all data are representative of at least three independent experiments. All data are presented as mean ± SD except for mouse tumor radiance quantification and body weight data shown as mean ± SEM. Significant differences were analyzed by one-way analysis of variance, two-way analysis of variance, student t, or log-rank test. *P*-values are represented as either not significant (ns), *P < 0.05, **P < 0.01, ***P < 0.001, or ****P < 0.0001.

## Supplementary information

Supplementary materials

## Data Availability

The datasets generated during and/or analyzed during the current study are available from the corresponding author on reasonable request.
