# Supplementary material for: T cells expressing CD5/CD7 bispecific chimeric antigen receptors with fully human heavy-chain-only domains mitigate tumor antigen escape
Source: Signal Transduct Target Ther. 2022 Mar 25;7:85. doi: 10.1038/s41392-022-00898-z (PMC8948246; doi:10.1038/s41392-022-00898-z)
Supplement: Supplementary file 1 — Supplementary materials [file 41392_2022_898_MOESM1_ESM.docx]

**Supplementary Materials for**

**T cells expressing CD5/CD7 bispecific chimeric antigen receptors with fully human heavy-chain-only domains mitigate tumor antigen escape**

Zhenyu Dai^1*^, Wei Mu^1*^, Ya Zhao^2^, Jiali Cheng^1^, Haolong Lin^1^, Kedong Ouyang^2^, Xiangyin Jia^2^, Jianwei Liu^2^, Qiaoe Wei^2^, Meng Wang^2^, Chaohong Liu^3^, Taochao Tan^2&^, Jianfeng Zhou^1&^

^1^Department of Hematology, Tongji Hospital, Tongji Medical College, Huazhong University of Science and Technology, Wuhan, Hubei 430030, China

^2^ Nanjing IASO Biotherapeutics, Nanjing, Jiangsu 210000, China

^3^ Department of Pathogen Biology, School of Basic Medicine, Tongji Medical College, Huazhong University of Science and Technology, Wuhan, China

* These authors contributed equally to this work.

Correspondence to: [jfzhou@tjh.tjmu.edu.cn](mailto:jfzhou@tjh.tjmu.edu.cn), [taochao.tan@iasobio.com](mailto:taochao.tan@iasobio.com)

**This file includes:**

Supplementary Figures 1 to 12

Supplementary Tables 1 to 2


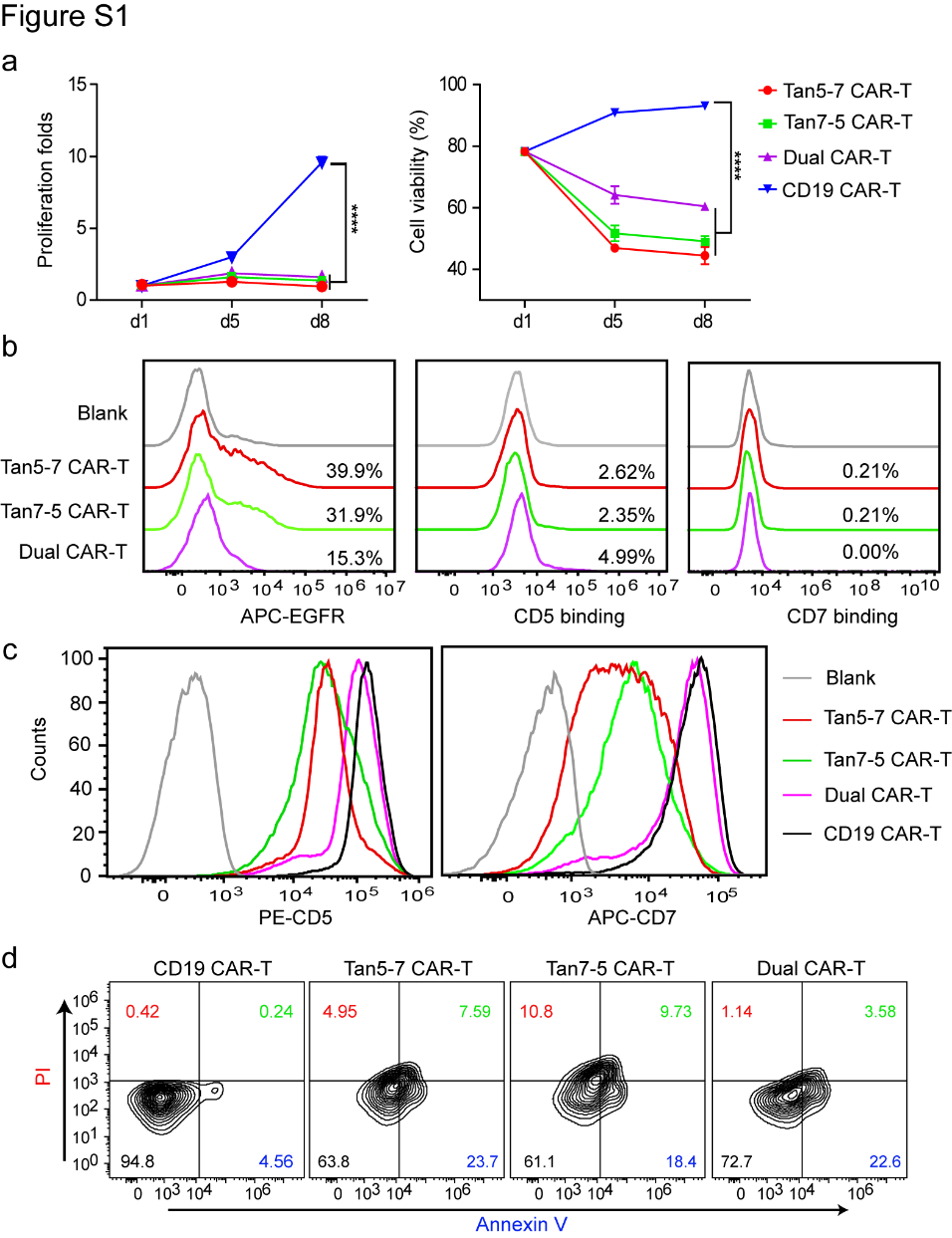


Figure S1. Fratricide of CD5/CD7 bispecific CAR-T cells. (a) The proliferation and cell viability of bispecific CAR-T cells. (b) Flow cytometry analysis of CAR expression on bispecific CAR-T cells measured by EGFRt antibody, recombinant CD5 and CD7 proteins on day 7. (c) The expression intensity of CD5 and CD7 molecules on bispecific CAR-T cells on day 7. (d) Expression of apoptosis-related markers on bispecific CAR-T cells on day 7.


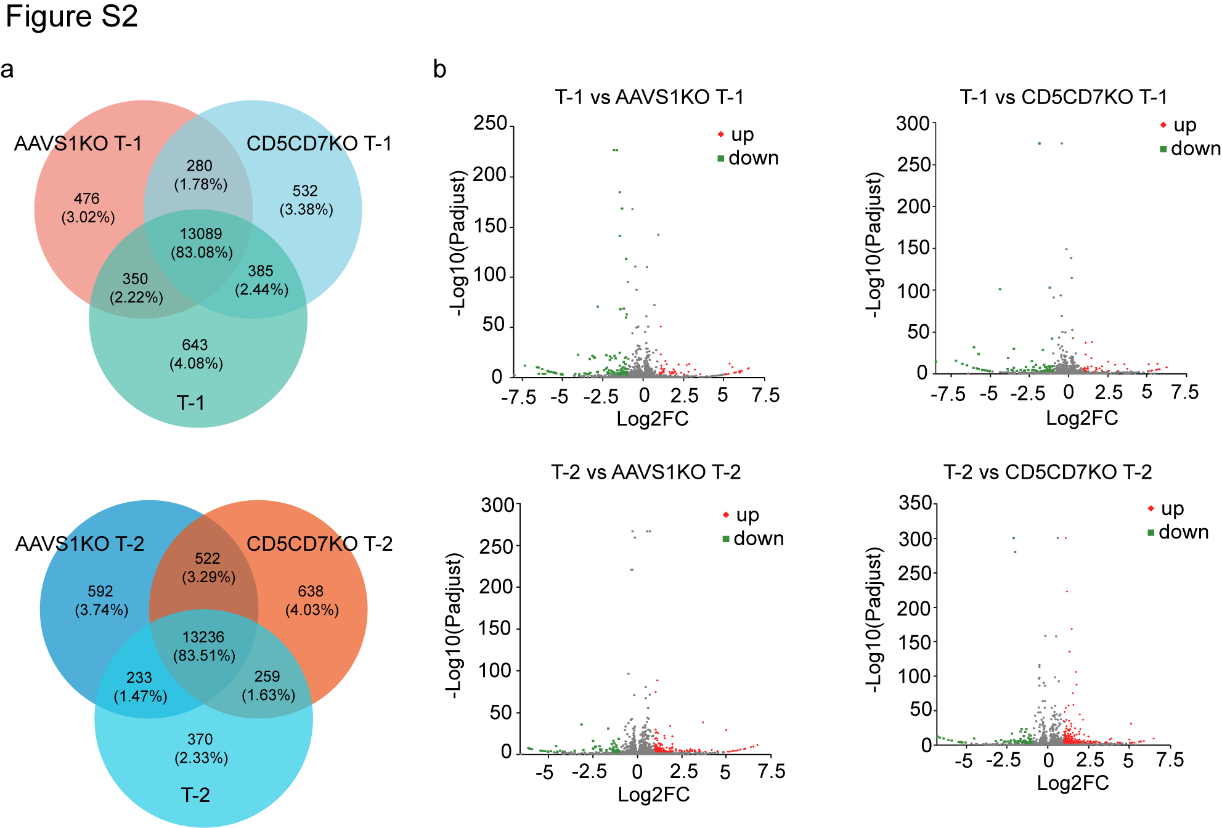


Figure S2. Transcriptomic profiles between T cells, AAVS1KO T cells, and CD5CD7KO T cells. (a) Venn diagram of the transcriptomic profiles in T cells, AAVS1KO T cells, and CD5CD7KO T cells of two donors. (b) Upregulated and downregulated genes in two donors showed by Volcano plots.


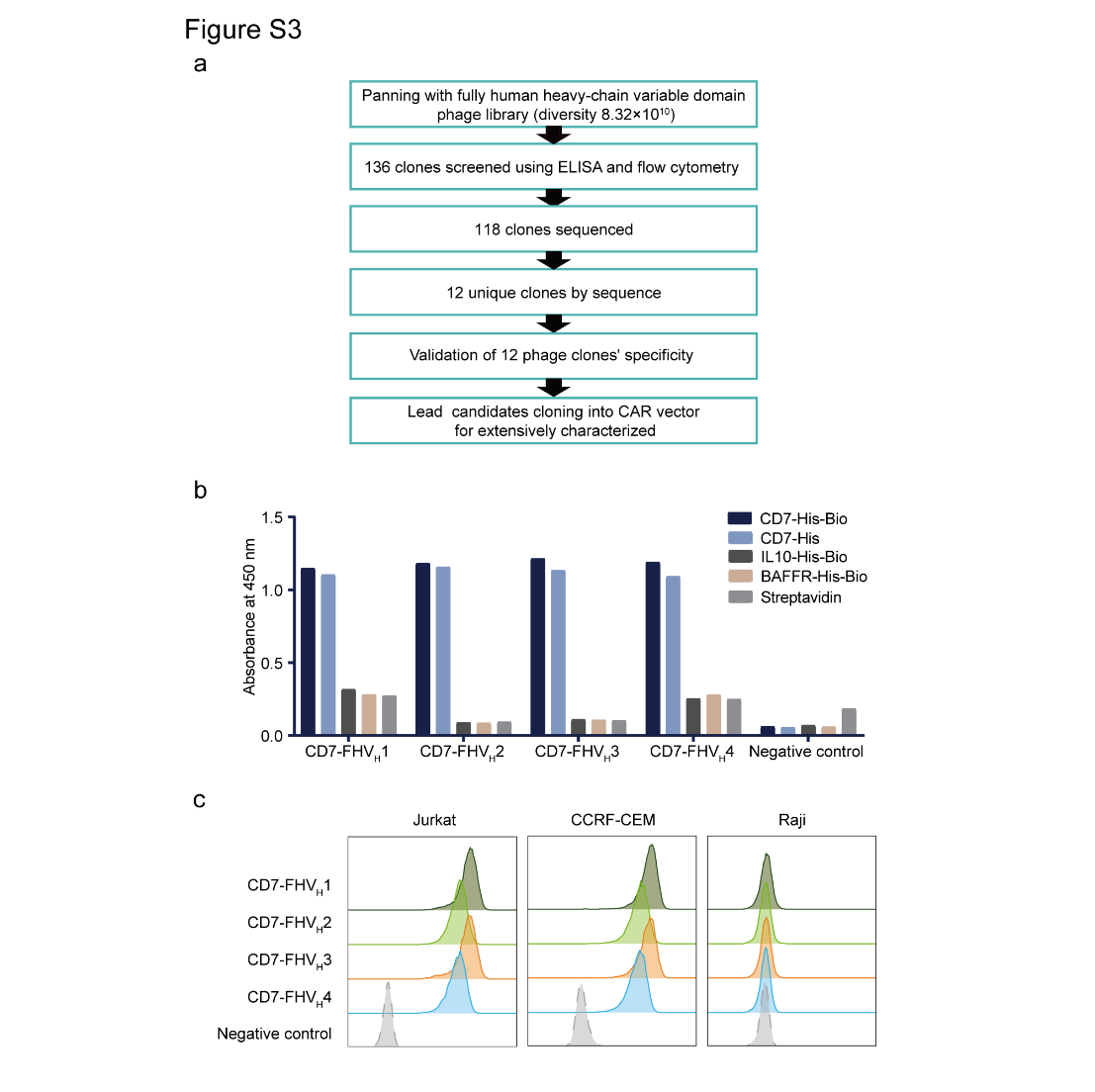


Figure S3. Identification of fully human (FH) CD7-specific heavy-chain variable domain (V_H_) using phage display library. (a) Illustration of the CD7-specific V_H_ development process. (b) Phage clones FHV_H_1–4 antibody binding capacity was determined after staining with anti-M13 bacteriophage coat protein g8p antibody and horseradish-peroxidase (HRP)-goat anti-mouse IgG antibody. M13KO7 phage (helper phage) was used as the negative control. (c) Phage clones FHV_H_1–4 were screened for binding to Jurkat and CCRF-CEM (both CD7^+^) and Raji (CD7^–^) cells, M13KO7 phage (helper phage) was regarded as the negative control, analyzed by flow cytometry.


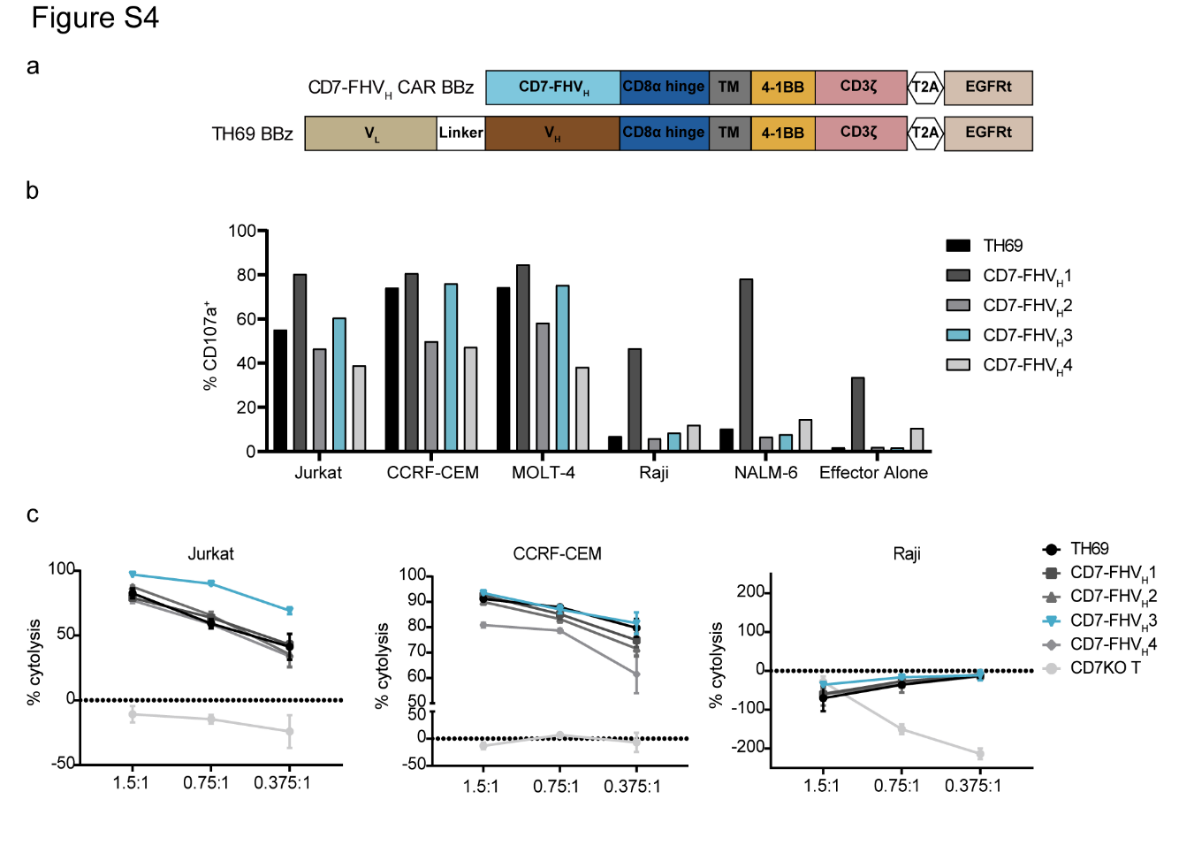


Figure S4. Functional comparison of fully human (FH) CD7-specific heavy-chain variable domain (V_H_) CARs. (a) Anti-CD7 phage clones FHV_H_1–4 and TH69 as a control were constructed into the CAR BBz lentiviral vector. (b) FHV_H_1–4 and TH69 CAR exhibited different levels of CD107a releasement after being stimulated by target cells. (c) Luciferase-based cytotoxicity assays of FHV_H_1–4 and TH69 CAR-T cells against tumor cells after 24 h incubated with target cells at indicated E:T ratios. Data are mean ± SD, three independent experiments were performed.


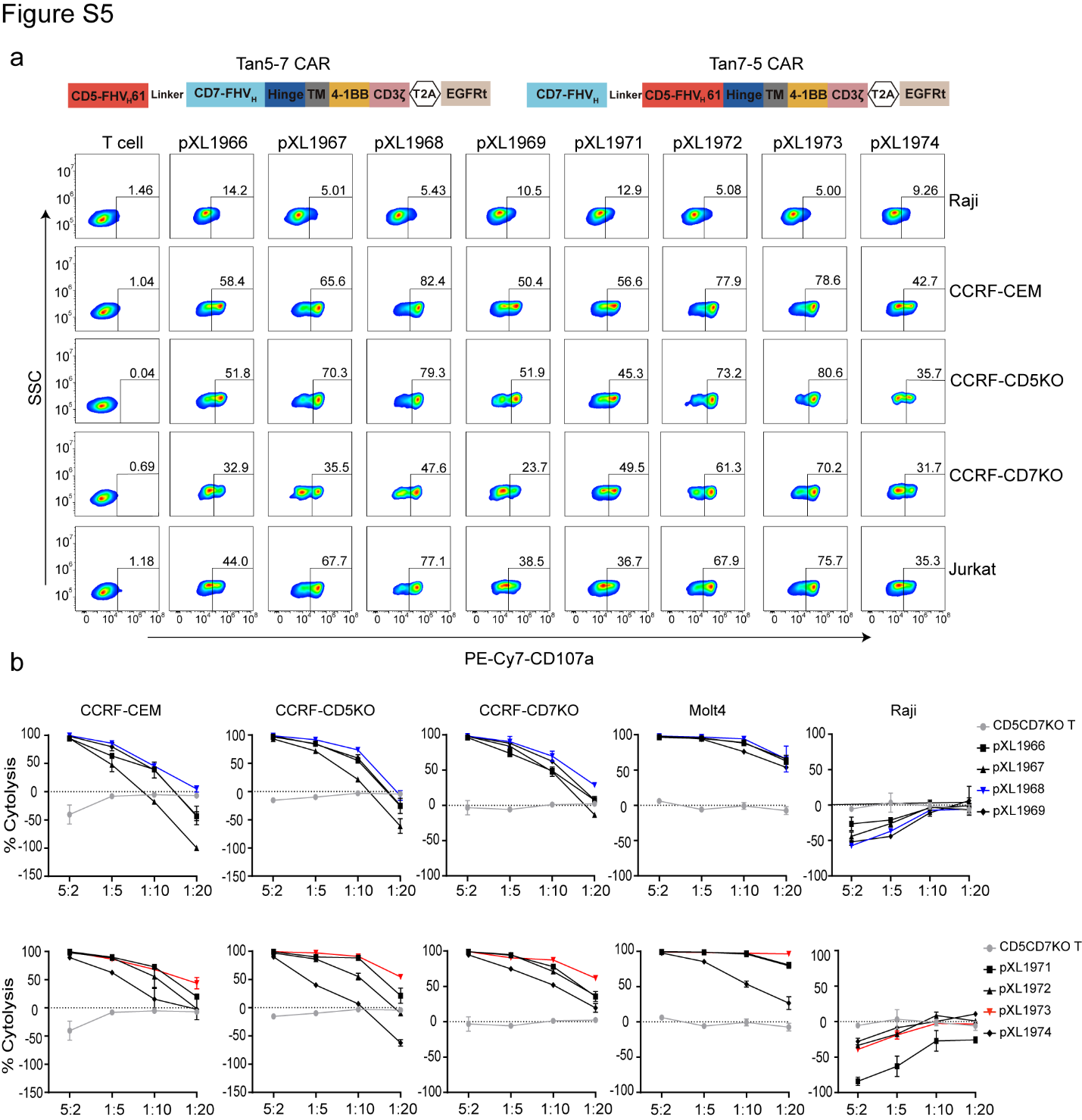


Figure S5. Functional comparison of Tan5-7 and Tan7-5 CARs. (a) A representative flow cytometric plot of CD107a staining (gated on CD8^+^ EGFR^+^ cells). CD7-FHV_H_s-CD5-FHV_H_61 CAR-T cells (upper panel) and CD5-FHV_H_61- CD7-FHV_H_s CAR-T cells (down panel) were stimulated with indicated target cells. pXL1966: CD7-FHV_H_1-CD5-FHV_H_61, pXL1967: CD7-FHV_H_2-CD5-FHV_H_61, pXL1968: CD7-FHV_H_3-CD5-FHV_H_61, pXL1969: CD7-FHV_H_4-CD5-FHV_H_61, pXL1971: CD5-FHV_H_61-CD7-FHV_H_1，pXL1972: CD5-FHV_H_61-CD7-FHV_H_2，pXL1973: CD5-FHV_H_61-CD7-FHV_H_3，pXL1974: CD5-FHV_H_61-CD7-FHV_H_4. (b) Luciferase-based cytotoxicity assays of CAR-T cells against tumor cells expressing CD5 and/or CD7 after 24 h incubated with target cells at indicated ratio. The data indicate mean ± SD from three independent experiments.


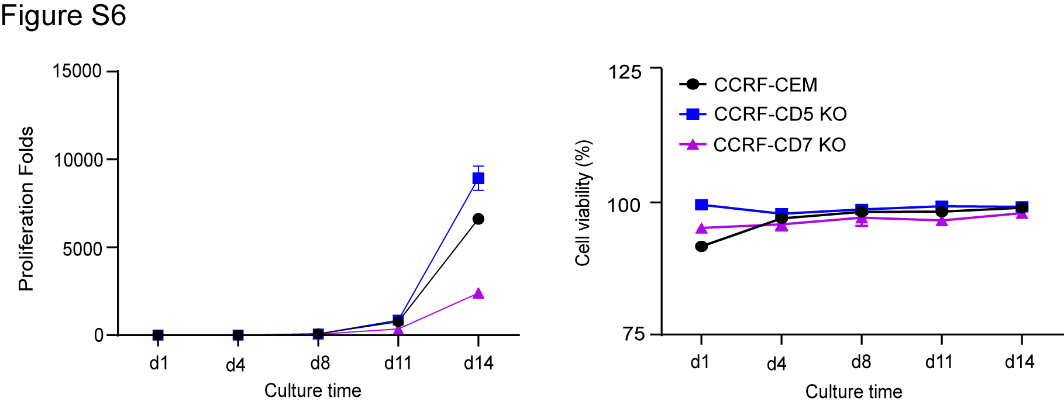


**Figure S6. Biological characteristics of CD5 or CD7 knockout CCRF-CEM cell line.** The proliferation and viability of CCRF-CEM, CCRF-CD5KO, and CCRF-CD7KO cells *in vitro*.


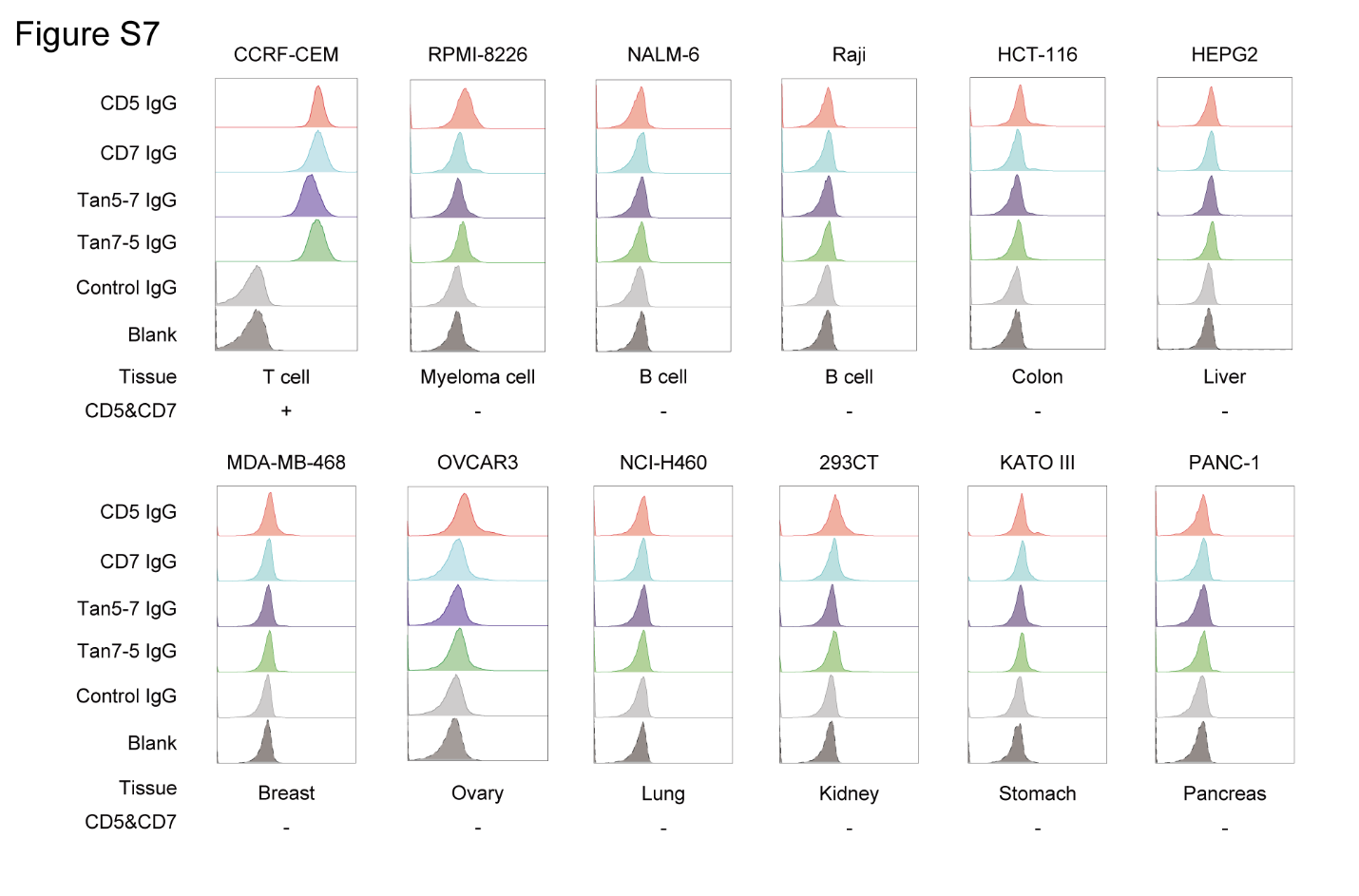
**Figure S7.** **Binding verification of antibodies to different tissues-derived CD5^-^CD7^-^ cell lines.** Different tissues-derived CD5^−^CD7^−^ cell lines and CD5^+^CD7^+^ CCRF-CEM cells were stained with CD5 (FHV_H_61) IgG, CD7 (FHV_H_3) IgG, Tan5-7 (CD5-FHV_H_61-CD7-FHV_H_3) IgG, Tan7-5 (CD7-FHV_H_3-CD5-FHV_H_61) IgG, and negative control IgG, respectively, followed by an APC-labeled anti-human IgG antibody then analyzed using flow cytometry.


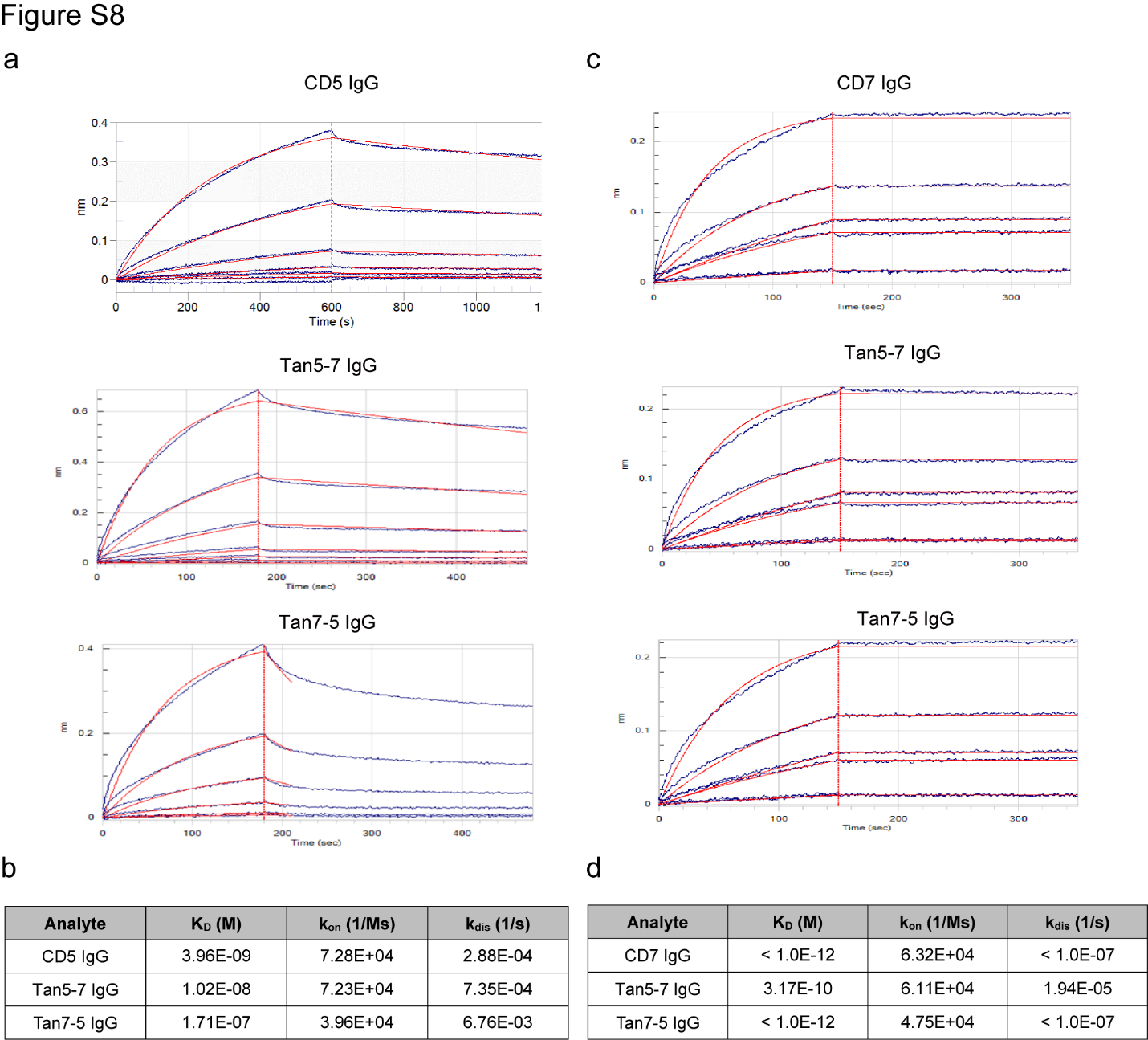


Figure S8. Binding affinity measurement of CD5 (FHV_H_61) IgG, CD7 (FHV_H_3) IgG, Tan5-7 (CD5-FHV_H_61-CD7-FHV_H_3) IgG, and Tan7-5 (CD7-FHV_H_3-CD5-FHV_H_61) IgG to CD5 and CD7 antigens. (a) The affinity between CD5 IgG, Tan5-7 IgG, Tan7-5 IgG, and recombinant human CD5 was detected using the bio-layer interferometry kinetics assay. (b) Tabulated kinetic and equilibrium dissociation constants (K_D_’s) of CD5 IgG, Tan5-7 IgG, and Tan7-5 IgG. (c) The affinity between CD7 IgG, Tan5-7 IgG, Tan7-5 IgG, and recombinant human CD7 was detected using the bio-layer interferometry kinetics assay. (d) Tabulated kinetic and equilibrium dissociation constants (K_D_’s) of CD7 IgG, Tan5-7 IgG, and Tan7-5 IgG.


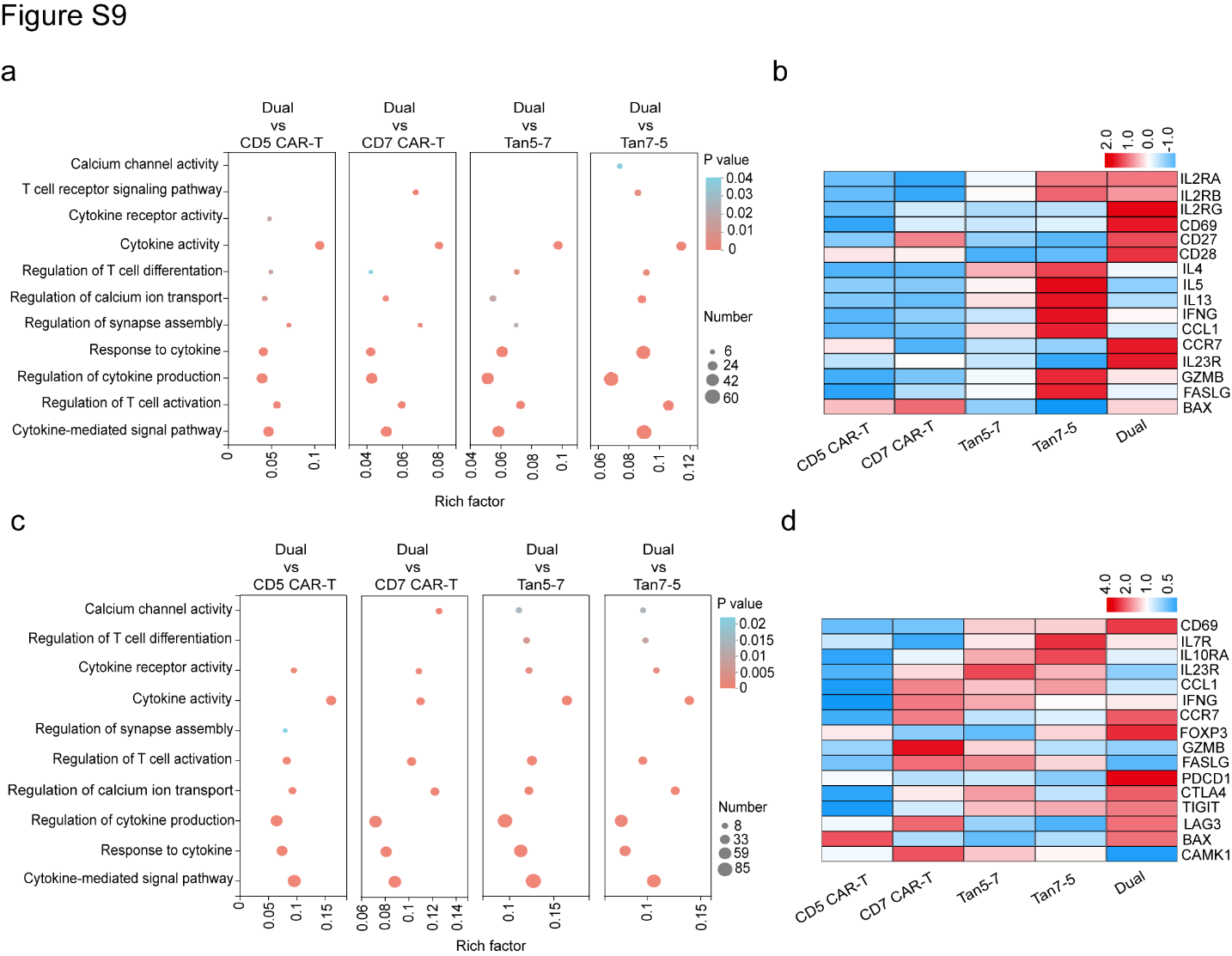


Figure S9. Transcriptional profiles of CD5 CAR, CD7 CAR, Tan5-7, Tan7-5, and Dual CAR-T cells exposed to the antigens. (a) Enriched gene sets in CAR-T cells related to T cell functional features after cocultured with mitomycin C-treated CCRF-CEM cells for 24h. (b) Heat map of selected genes related to T cell activation, cytokine production, and cytolysis with differential expression. (c) Enriched gene sets in CAR-T cells related to T cell functional features after stimulated by mitomycin C-treated CCRF-CEM cells by 4 rounds. (d) Heat map of selected genes related to T cell exhaustion, cytokine production, and cytolysis with differential expression.


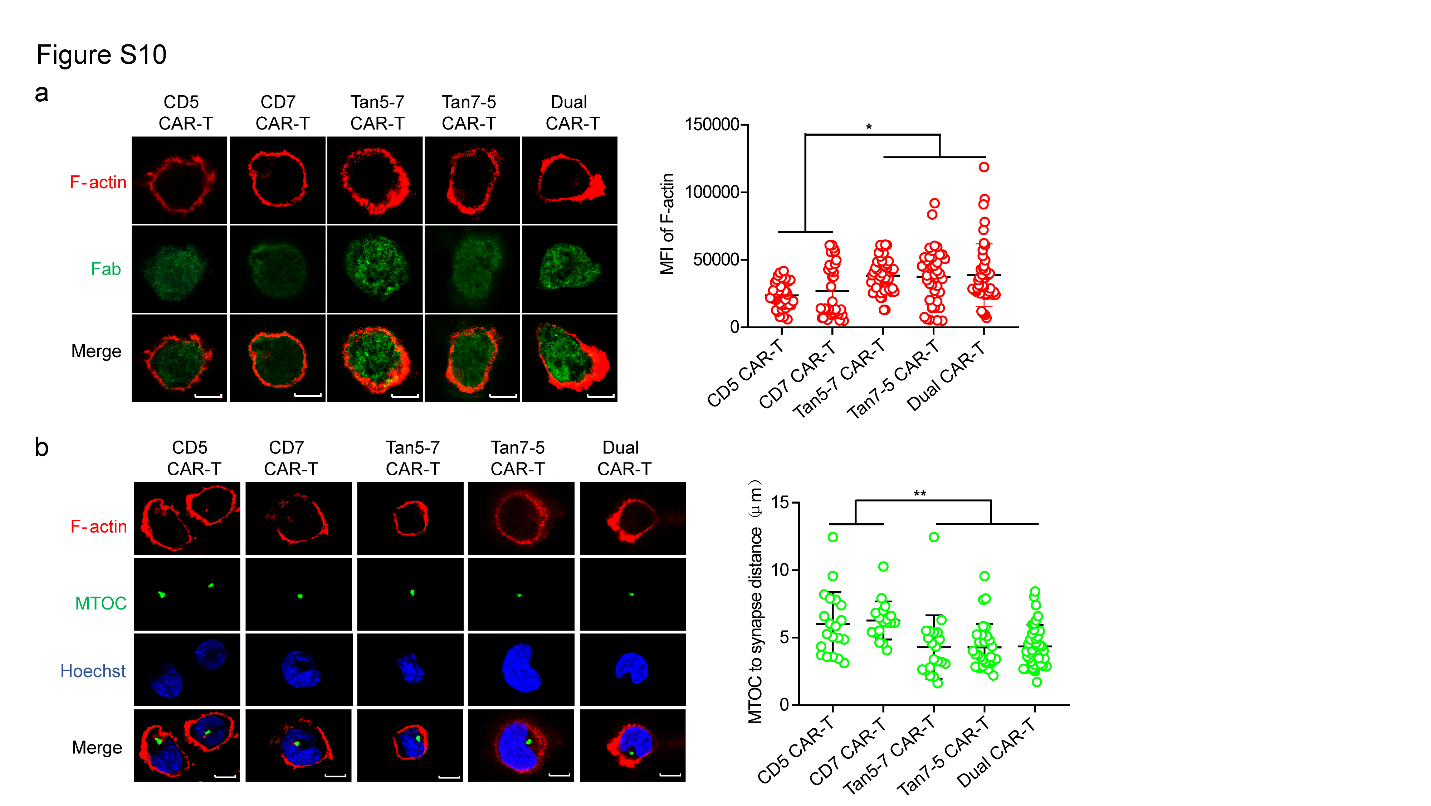


Figure S10. CD5/CD7 bispecific CAR-T cells formed stable IS than CD5 CAR or CD7 CAR-T cells. (a) Representative confocal imaging of synapses formed by CAR-T cells. CD5 CAR, CD7 CAR, Tan CARs, and Dual CAR-T cells were loaded onto CD5- and CD7-coated lipid bilayers for 15 minutes, fixed and stained for CAR (green) and F-actin (red). (b) Quantification of IS structures on the lipid bilayer-T cell focal plane by measuring the MFIs of F-actin. Error bars show ±SD. *P* values were calculated with an unpaired t-test. (c) Representative MTOC confocal imaging of indicated CAR-T cells. CD5 CAR, CD7 CAR, Tan CARs, and Dual CAR-T cells were loaded onto CD5- and CD7-coated lipid bilayers for 15 minutes, fixed and stained for γ tubulin (green), F-actin (red), and Hoechst (blue). (d) MTOC polarization at the IS. For each group,18-38 random cells were evaluated. *P* values were calculated with an unpaired t-test. Scale bars, 5 μm.


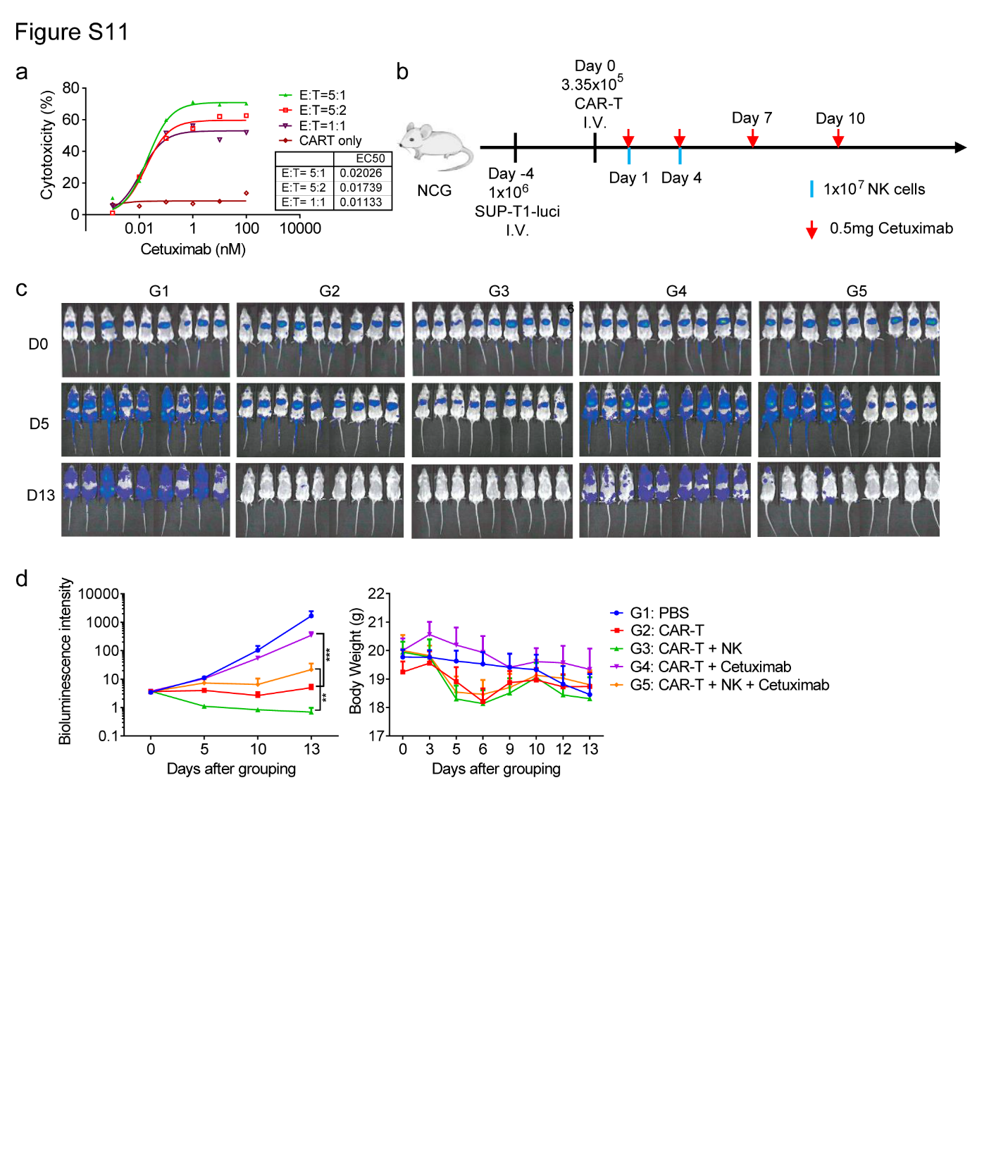


Figure S11. Cetuximab efficiently depletes EGFRt expressing anti-CD5 CAR-T cells *in vitro* and in NCG mice. (a) NK cells and EGFRt expressing anti-CD5 CAR-T cells were incubated at the ratio of 5:1, 5:2, 1:1, and indicated Cetuximab concentration. After 4 hours of coculture, cytolysis of NK cells mediated by Cetuximab was measured by FACS. Three independent experiments were performed, and representative results are shown. (b) Experimental schema to evaluate the depletion effect of cetuximab after anti-CD5 CAR-T cells infusion into NCG mice. Cyan line: 1×10^7^ NK cells i.v.; Red arrow: Cetuximab i.v.; (c) Growth and staging of the tumor monitored by bioluminescence imaging. (d) Quantification of tumor burden and body weight of different experimental groups.


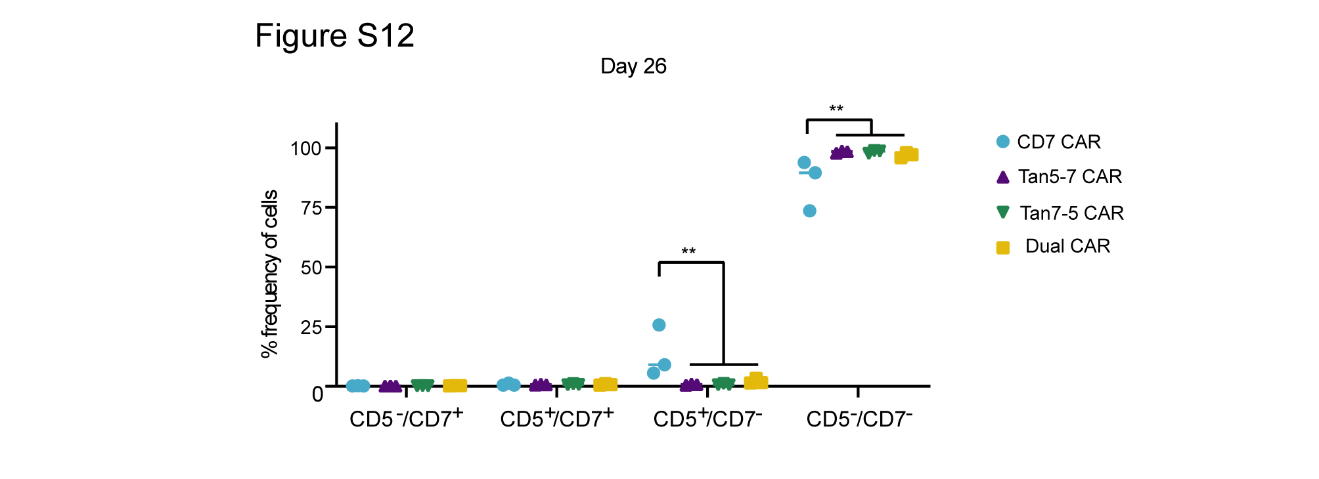


Figure S12. Flow cytometry analysis of CD5 and CD7 expression in peripheral blood of NCG mice in the CCRF-CEM MIX tumor model. To study the expression of CD5 and CD7 on the surface of CD45^+^ cells in the peripheral blood of NCG mice after CAR-T treatment, the expression of CD5 and CD7 in the peripheral blood of mice in the CD7 CAR, Tan5-7 CAR, Tan7-5 CAR, and Dual CAR-T treated groups was analyzed via flow cytometry on day 26. Only one mouse in the CD5 CAR-T treated group survived on day 26, so it was not included in this comparison.

**Table S1. CD5 and CD7 guide RNA sequences in the study**

| Name | Sequence |
| --- | --- |
| CD5 gRNA-7 | CGGCTCAGCTGGTATGACCCG |
| CD7 gRNA-85^3^ | GGAGCAGGTGATGTTGACGGG |

**Table S2. Phage protein panning**

| FHV_H_ phage antibody library | Round | Strategy | Recovery rates | Enrichment |
| --- | --- | --- | --- | --- |
| XL-SD-1-2004-2 | 1st | CD7 protein | 3.66E-05 | / |
|  | 2nd | CD7 protein | 1.49E-04 | 4.07 |
|  | 3rd | CD7 protein | 1.47E-02 | 98.66 |
| XL-VH-2004-2 | 1st | CD7 protein | 7E-05 | / |
|  | 2nd | CD7 protein | 1.6E-04 | 2.29 |
|  | 3rd | CD7 protein | 3E-03 | 18.75 |
| XL-VH-M2 | 1st | CD7 protein | 7.66E-05 | / |
|  | 2nd | CD7 protein | 1.73E-04 | 2.26 |
|  | 3rd | CD7 protein | 7.45E-03 | 43.06 |

**REFERENCE**

1 Mu, W. *et al.* In vitro transcribed sgRNA causes cell death by inducing interferon release. *Protein Cell* **10**, 461-465 (2019).

2 Tong, C. *et al.* Optimized tandem CD19/CD20 CAR-engineered T cells in refractory/relapsed B-cell lymphoma. *Blood* **136**, 1632-1644 (2020).

3 Gomes-Silva, D. *et al.* CD7-edited T cells expressing a CD7-specific CAR for the therapy of T-cell malignancies. *Blood* **130**, 285-296 (2017).
